# Disruption of the β-catenin destruction complex via Ephexin1-Axin1 interaction promotes colorectal cancer proliferation

**DOI:** 10.1038/s12276-024-01381-1

**Published:** 2025-01-01

**Authors:** Jeeho Kim, Young Jin Jeon, In-Youb Chang, Jung-Hee Lee, Ho Jin You

**Affiliations:** 1 Laboratory of Genomic Instability and Cancer Therapeutics, Gwangju, South Korea; 2 Department of Pharmacology, Gwangju, South Korea; 3 Department of Anatomy, Gwangju, South Korea; 4 Department of Cellular and Molecular Medicine, Chosun University School of Medicine, 375 Seosuk-dong, Gwangju, 501-759 South Korea

**Keywords:** Colon cancer, Oncogenes

## Abstract

Wnt signaling is essential for cell growth and tumor formation and is abnormally activated in colorectal cancer (CRC), contributing to tumor progression; however, the specific role and regulatory mechanisms involved in tumor development remain unclear. Here, we show that Ephexin1, a guanine nucleotide exchange factor, is significantly overexpressed in CRC and is correlated with increased Wnt/β-catenin pathway activity. Through comprehensive analysis, including RNA sequencing data from TCGA and functional assays, we observed that Ephexin1 promotes tumor proliferation and migration by activating the Wnt/β-catenin pathway. This effect was mediated by the interaction of Ephexin1 with Axin1, a critical component of the β-catenin destruction complex, which in turn enhanced the stability and activity of β-catenin in signaling pathways critical for tumor development. Importantly, our findings also suggest that targeting Ephexin1 may increase the efficacy of Wnt/β-catenin pathway inhibitors in CRC treatment. These findings highlight the potential of targeting Ephexin1 as a strategy for developing effective treatments for CRC, suggesting a novel and promising approach to therapy aimed at inhibiting cancer progression.

## Targeting Ephexin1 enhances Wnt inhibitor efficacy in CRC

Wnt signaling is crucial for cell growth and is often overactive in cancers like colorectal cancer (CRC). This study explores the role of a protein called Ephexin1 in CRC. Researchers found that Ephexin1 is overexpressed in CRC and promotes tumor growth by activating Wnt/β-catenin signaling. They used various cell lines and animal models to study this interaction. The study involved analyzing gene expression data from The Cancer Genome Atlas (TCGA) and conducting experiments to understand how Ephexin1 affects β-catenin stability, a key player in Wnt signaling. Results showed that reducing Ephexin1 levels made CRC cells more sensitive to Wnt inhibitors, suggesting that targeting Ephexin1 could enhance treatment efficacy. The researchers conclude that Ephexin1 is a potential therapeutic target for CRC, as it plays a significant role in Wnt signaling modulation.

This summary was initially drafted using artificial intelligence, then revised and fact-checked by the author.

## Introduction

Wnt signaling plays a pivotal role in regulating various biological processes, including cell proliferation, differentiation, tissue regeneration, and tumorigenesis [1–3]. It serves primarily as a growth-stimulating factor that promotes cell proliferation [4]. This signaling pathway is notably upregulated in several cancers, with colorectal cancer (CRC) being a prime example where hyperactivation of Wnt signaling is a key contributor [5–7]. Despite the recognized importance of Wnt/β-catenin signaling in cancer progression, there are currently no clinically approved therapies targeting this pathway. However, the established importance of Wnt/β-catenin signaling in cancer has prompted the development of numerous therapies designed to block this pathway [8–10].

Wnt signaling can be categorized into two pathways: β-catenin-dependent (canonical) and β-catenin-independent (noncanonical) signaling [11, 12]. The canonical pathway plays a critical role in regulating β-catenin levels. In the absence of a Wnt ligand, β-catenin is maintained at low levels through ubiquitin-dependent proteasome degradation, which is mediated by the β-catenin destruction complex comprising proteins such as Axin1, adenomatous polyposis coli (APC), casein kinase 1 (CK1), and Glycogen Synthase Kinase 3β (GSK3β) [13–16]. Axin1 is crucial for the complex, facilitating β-catenin phosphorylation by CK1 and GSK3β, leading to its ubiquitination by E3 ligases such as SCF^βTrCP^ and subsequent degradation [14, 15, 17, 18]. Despite extensive research, the detailed molecular processes involved in tumor formation driven by Wnt/β-catenin signaling and how to effectively target this pathway are still not fully understood.

Ephexin1, a member of the Dbl family of guanine nucleotide exchange factors (GEFs), plays a significant role in neurophysiological processes through its involvement in Ephrin signaling. Ephexin1 is found mainly in the developing nervous system and is minimally present in other organs [19, 20]. The overexpression of oncogenic K-Ras is linked to the upregulation of Ephexin1 [21, 22], and its expression levels increase in association with the progression of lung cancer (LC), colorectal (CRC), and thyroid cancers [22–24]. Notably, the absence of Ephexin1 in LC and CRC leads to reduced apoptosis and migration [22]. In addition to its role in Ephrin signaling, Ephexin1 likely contributes to the Wnt/β-catenin signaling pathway. This relationship is reinforced by the involvement of other GEF family proteins, such as p114-RhoGEF and GEF-H1, in Wnt/β-catenin signaling [25]. Inhibition of this pathway, either through the removal of the Wnt coreceptors LRP5/LRP6 or by the use of sclerostin, results in reduced Ephexin1 levels [26, 27]. These observations support the potential involvement of Ephexin1 in the Wnt/β-catenin signaling pathway, although its exact functional role remains to be fully elucidated.

In the present study, we found that Ephexin1 is significantly overexpressed in CRC and promotes tumor growth by activating the Wnt/β-catenin pathway. Analysis of TCGA data revealed a strong link between Ephexin1 expression and Wnt/β-catenin activation in CRC. The interaction of Ephexin1 with Axin1 affected β-catenin stability and Wnt signaling, indicating that Ephexin1 could be a valuable target for enhancing the efficacy of Wnt pathway inhibitors in CRC treatment. These findings highlight the critical role of Ephexin1 in Wnt signaling modulation and its therapeutic potential in CRC.

## Materials and methods

### Cell culture and transfection

The normal cell lines CCD18co and CCD841coN were cultured in MEM (Invitrogen, Carlsbad, CA, USA). A549, H23, H358, H1299, H1666, HCC827, H1650, LoVo, HCT15, and HCT116 cells were grown in RPMI-1640 medium (Invitrogen). SK-MES-1, Calu-3, Caco-2, and LS174T cells were cultured in MEM, while HEK293T, SW480, SW620, DLD-1, and HT-29 cells were maintained in Dulbecco’s modified Eagle’s medium (DMEM) (Invitrogen). All cell lines were acquired from the American Type Culture Collection (ATCC; Manassas, VA, USA). The media were supplemented with 10% fetal bovine serum (FBS) and 1% penicillin‒streptomycin antibiotic solution. The cells were incubated at 37 °C in a 5% CO_2_ humidified atmosphere. The plasmids were transiently transfected into mammalian cells using TurboFect in vitro Transfection Reagent (Thermo Scientific, Waltham, MA, USA). IWR1-endo (Cat. No. S67086) was obtained from Selleckchem (Houston, TX, USA).

### Plasmid constructs and cloning

The full-length and serial deletion constructs of human Ephexin1 have been previously described [22]. Human Axin1 was amplified from HEK293T cells by RT-PCR, and subsequently cloned and inserted into the pCI-neo-Flag or pCI-neo-V5 mammalian expression vector (Promega, Madison, WI, USA). To prepare serial deletion constructs of Axin1 (ΔRGS, ΔRGS/p53, ΔRGS/p53/GSK3β, RNF11/DIX, ΔDIX, RNF11, p53, RGS, Δp53, ΔRNF11/DIX, ΔRNF11, GSK3β/β-catenin, GSK3β/β-catenin/DIX, and Δp53/RNF11), the PCR products were cloned and inserted into the XhoI-NotI or XhoI-HindIII sites of the pCI-Flag vector. All the constructs were verified by DNA sequencing. For the isolation of recombinant proteins, the GST-Ephexin1 construct (full-length or DH/PH domain) was constructed as previously described, and Hisx6-Axin1 (RGS or DIX domain) was cloned and inserted into the pET28a vector (Novagen). A comprehensive list of all the PCR primers used in this study is provided in Supplementary Table 1.

### RNAi and stable Ephexin1-knockdown cells

The cells were transfected with siRNAs (40 nM) using Lipofectamine RNAi max (Invitrogen). After 36 h, the cells were trypsinized, replated, and subjected to a second round of transfection for another 36 h. The knockdown efficiency was confirmed by Western blot analysis. The sequences of the Ephexin1 siRNAs and shRNAs have been previously described [22, 24]. siRNA and DNA oligonucleotide were synthesized by Macrogen (Seoul, South Korea).

### Immunoblot and immunoprecipitation analysis

Cell extracts were prepared using IP150 lysis buffer (20 mM Tris-HCl pH 7.6, 150 mM NaCl, 0.5% Nonidet P-40, and 10% glycerol) containing protease inhibitors (1 mM Na_2_VO_4_, 10 mM NaF, 2 mM PMSF, 5 μg/ml leupeptin, 10 μg/ml aprotinin, and 1 μg/ml pepstatin A) (Roche, Switzerland). Equal amounts of protein were separated by SDS‒PAGE and transferred onto PVDF membranes (PALL Life Sciences, USA). The membranes were then incubated with appropriate primary antibodies overnight at 4 °C, followed by incubation with horseradish peroxidase-conjugated secondary antibodies for 1 hour at room temperature. The protein bands were visualized via an enhanced chemiluminescence (ECL) detection system (iNtRON Biotechnology, Korea). For the immunoprecipitation of protein complexes, cell extracts were precleared with protein G-Sepharose beads (GE Healthcare) and then incubated with specific antibodies. The immune complexes were analyzed by immunoblotting with corresponding antibodies. A complete list of the antibodies used can be found in Supplementary Table 2.

### Cell growth assay

The cell growth assay was performed using an MTT assay. An equal number of HCT116 cells were seeded in triplicate in each well of 48-well plates at a density of 1 × 10^4^ cells/0.2 ml/well. Twenty microliters of MTT solution (5.0 mg/ml) in RPMI-1640 medium was added to each well, and the plates were incubated for the indicated times at 37 °C. The purple formazan crystals that formed were dissolved in 200 μl of MTT solvent (0.1% NP-40 and 4 mM HCl in isopropanol) by gentle mixing at room temperature. The optical densities of the wells were measured at 570 nm using a microplate spectrophotometer (Epoch, BioTek, Winooski, VT, USA).

### Soft agar colony formation assay

Soft agar assays were conducted in six-well plates, each containing a base layer of 2 ml of medium (at a final concentration of 1X) mixed with 0.6% low-melting point agarose (Duchefa Biochemie, Netherlands). The plates were chilled at 4 °C until the medium solidified. A growth layer consisting of 2 ml of 1X medium combined with 0.3% low-melting point agarose and 1 × 10^4^ cells was subsequently added. The plates were again chilled at 4 °C until the growth layer solidified. An additional 1 ml of 1X medium without agarose was gently layered on top of the growth layer. The cells were incubated at 37 °C in a 5% CO_2_ atmosphere for ~14–21 days. Colonies were then stained with 0.005% crystal violet (Sigma‒Aldrich) and counted. Images were analyzed using an Olympus microscope (Olympus, Tokyo, Japan) and Image-Pro Plus 4.5 software (Media Cybernetics Inc., Rockville, MD, USA). The assays were performed in triplicate.

### Cell migration assay

In vitro cell migration assays were conducted using a 24-well transwell plate with 8-μm polyethylene terephthalate membrane filters (BD Biosciences) to separate the lower and upper culture chambers. The cells were cultured until they reached subconfluence (75–80%) and then serum starved for 24 h. After detachment with trypsin, the cells were washed with PBS and resuspended in a serum-free medium, and a suspension of 2 × 10^4^ cells was added to the upper chamber. A complete medium was added to the lower chamber. The cells that had not migrated were removed from the upper surface of the filters using cotton swabs. In contrast, cells that had migrated to the lower surface were fixed with 4% formaldehyde and stained with 0.2% crystal violet. Images of three random fields, magnified 10x, were captured from each membrane, and the number of migratory cells was counted. The mean of the triplicate assays for each experimental condition was calculated.

### Tumor formation in nude mice

The mice utilized in this study were 6-week-old male BALB/c nude mice acquired from NARA Biotech (Seoul, Korea). They were housed in our pathogen-free facility and managed according to standard use protocols and animal welfare regulations. HCT116 cells were harvested, resuspended in PBS, and then 1 × 10^6^ HCT116 cells were injected subcutaneously into both the left and right flanks of the mice. Once the tumors became visible, their size was measured at 3–4-day intervals using micrometer calipers. Tumor volumes were calculated using the following formula: volume = 0.5 × a × b^2^, where “a” and “b” represent the larger and smaller tumor diameters, respectively. Approximately 3 weeks postinjection, the mice were humanely sacrificed, and the primary tumors were excised and immediately weighed.

### Immunostaining

Immunohistochemistry was conducted on tissue microarrays of colorectal cancer samples. Tissue microarrays, which represent cancer samples of various grades and adjacent normal tissues, were acquired from Super Bio Chips (CDA3) (Seoul, South Korea). For immunohistochemistry, heat-induced antigen retrieval was performed using 1X antigen retrieval buffer (pH 9.0) (Abcam) at 95 °C for 15 min. Following quenching of endogenous peroxidase activity and blocking in a 3% H_2_O_2_ solution, the tissues were incubated with the following primary antibodies: anti-Ephexin1 (PA5-52521, Thermo Scientific), anti-Lgr5 (MA5-25644, Thermo Scientific), and anti-β-catenin (#610154, BD) overnight at 4 °C. The tissues were then incubated with an HRP-conjugated secondary antibody for 1 h at room temperature and further incubation with 3,3’-diaminobenzidine (DAB) for 2 min. The slides were subsequently counterstained with Harris’s hematoxylin. The staining intensity was scored from 0 to 4, and the extent of staining was scored from 0 to 100%. A final quantitation score for each stain was determined by multiplying the intensity and extent scores. The slides were independently analyzed by two pathologists.

### Proximity ligation assay (PLA)

The proximity ligation assay (PLA) was conducted on tissue microarrays of colorectal cancer of various grades and adjacent normal tissues, which were acquired from Super Bio Chips (CDA3). The assay began with heat-induced antigen retrieval using 1X antigen retrieval buffer (pH 9.0) (Abcam) at 95 °C for 15 min, followed by blocking with Duolink™ blocking solution. The tissues were then incubated with primary anti-Ephexin1 (rabbit) and anti-Axin1 (mouse) antibodies overnight at 4 °C. The slides were subsequently incubated with anti-rabbit MINUS and anti-mouse PLUS PLA probes (Duolink™, Sigma‒Aldrich) for 1 h at 37 °C. This was followed by a 30-min incubation with ligation buffer and ligase (Duolink™, Sigma‒Aldrich) at 37 °C, and then, amplification buffer and polymerase (Duolink™, Sigma‒Aldrich) were added for an additional 120 min at 37 °C. The stained samples were analyzed using a fluorescence microscope (Nikon, Japan). Ephexin1-Axin1 PLA high and low values were based on the average value of the PLA score.

### Bioinformatics analysis using the cancer genome atlas (TCGA) databases

Data from The Cancer Genome Atlas (TCGA; https://www.cancer.gov/about-nci/organization/ccg/research/structural-genomics/tcga) were downloaded using the UCSC Xena browser Data Hub (https://xenabrowser.net/hub/). The RNA sequencing data, which were measured by Illumina HiSeq and RSEM normalization, were downloaded when available. The mRNA expression data from the TCGA discovery set were transformed into a log2 scale, and correlation analyses were visualized using GraphPad Prism (GraphPad Software Inc., CA, USA). *P* values between groups were calculated using Student’s *t*-test with GraphPad Prism.

### RNA sequencing analysis and GSEA

Total RNA was harvested directly from cell culture plates using 1 ml of TRIzol reagent per 60 mm plate. Total RNA was isolated and treated with DNase I (Invitrogen). RNA sequencing was performed using an Illumina NovaSeq 6000™ sequencer at DNA_Link™ (Seoul, Korea). The RNA-seq reads were initially mapped to the human genome GRCh37/hg19 build using Tophat version 2.0. 13 (http://ccb.jhu.edu/software/tophat/). The aligned results were analyzed with Cuffdiff version 2.2. 1 (http://cole-trapnell-lab.github.io/cufflinks/papers/) to calculate the FPKM values and report the differentially expressed genes. For library normalization and dispersion estimation, both geometric and pooled methods were utilized (http://cole-trapnell-lab.github.io/cufflinks/cuffdiff/). Scatter plots and heatmaps were created using the ‘heatmap’ function in the “ggplot” package in R version 3.4.1. The data discussed in this publication have been deposited in the NCBI Gene Expression Omnibus (GEO) and are accessible through GEO series accession number GSE220669. Gene set enrichment analysis (GSEA) was performed using the GSEA preranked module on the GSEA software (version 4.3.0), with log_2_-fold change values for the ranked genes.

### Identification of genes related to sensitivity to Wnt/β-catenin-targeting agents

Datasets of human cancer cell lines were obtained from The Cancer Dependency Map Project (DepMap, https://depmap.org/portal/, version 23Q2). Data regarding responses to Wnt/β-catenin-targeting agents, including ICG-001, IWR1-endo, niclosamide, salinomycin, WNT-C59, and XAV-939, were sourced from the drug sensitivity PRISM file (version 23Q2) [28]. The RNA expression data were obtained from the CCLE [29] RNA-seq gene expression data file (log_2_(TPM + 1)). Genome-wide RNAi loss-of-function screening data were derived from two large-scale CRISPR and RNAi experiments (CERES [30], Achilles [31], and DRIVE [32]). Gene effects were calculated using DEMETER2 [33] within DepMap. The p values obtained from these analyses were then converted to −log_10_ (*p* value) values to score each gene.

### Quantitative real-time PCR (RT‒qPCR)

Total RNA was extracted from cell lysates using TRIzol (Invitrogen), and 2 µg of total RNA was reverse transcribed to cDNA using oligo dT primers and M-MuLV Reverse Transcriptase (Invitrogen). RT‒qPCR analysis was performed using specific primers and the SYBR Premix Ex Taq™ kit (TaKaRa Bio, Shiga, Japan). The transcripts were detected via the CFX96 Real-Time PCR Detection System (Bio-Rad, CA, USA). The primers used for RT‒qPCR targeted Ephexin1, Wnt7a, Axin2, CXCL8, TERT, YWHAB, APC, DKK1, TCF7, Lgr5, Wnt9a, ID2, CSNK2B, PPP3CA, Cyclin D1, CHD1, ROCK2, XPO1, YWHAZ, FRAT2, TBL1XR1, PRKACB, HDAC1, and β-actin. Each sample was analyzed in triplicate, and target genes were normalized relative to the reference housekeeping gene β-actin. Relative mRNA expression levels were calculated using the comparative threshold cycle (Ct) method, with β-actin as the control, according to the following formula: ΔCt = Ct(β-actin) − Ct(target gene). The fold change in gene expression normalized to that of β-actin and relative to that of the control sample was calculated as 2^-^ΔΔC_t_. The RT‒qPCR primer sequences are listed in Supplementary Table 3.

### In vitro GST pulldown assay

Bacterially expressed GST-Ephexin1 (full-length or DH/PH domain) and GST alone were immobilized onto Glutathione Sepharose 4B beads (GE Healthcare) and incubated with bacterially expressed His\x6-Axin1 (RGS or DIX domain) fusion proteins overnight at 4 °C. The GST bead-bound complexes were then washed five times with GST lysis buffer (20 mM HEPES, pH 7.6; 150 mM NaCl; 5 mM MgCl_2_; 1% Triton X-100; and 5% glycerol), and the bound proteins were separated via SDS‒PAGE and analyzed by Western blotting with appropriate antibodies.

### Prediction of the Ephexin1, Axin1, and APC structures

To predict the structures of Ephexin1(1–457aa), Axin1(1–211aa) and APC (1567–1595aa, 1716–1734aa, and 2032–2050aa), the corresponding sequences were processed using AlphaFold-Multimer (https://github.com/deepmind/alphafold, Version 2.3.0) [34] encased in the ColabFold [35] package, which takes advantage of the MMseq2 server for automated MSA (Multiple Sequence Alignment) generation. The open-source PyMOL system (https://pymol.org/2/) was used for visualization.

### Statistical analysis

The data were presented as the means ± SEMs from three independent experiments. Significant differences between groups were assessed using a two-tailed paired Student’s *t*-test or two-way ANOVA with GraphPad Prism (GraphPad Software Inc., CA, USA). The results with values of *p < 0.05, ** *p* < 0.01, and *** *p* < 0.001 were considered statistically significant.

### Ethics statement

All the animal studies were reviewed and approved by the Institutional Animal Welfare and Use Committee of Chosun University School of Medicine (CIACUC2023-A0010).

## Results

### Ephexin1 is correlated with Wnt/β-catenin target gene expression in colorectal cancer

**Fig. 1:**
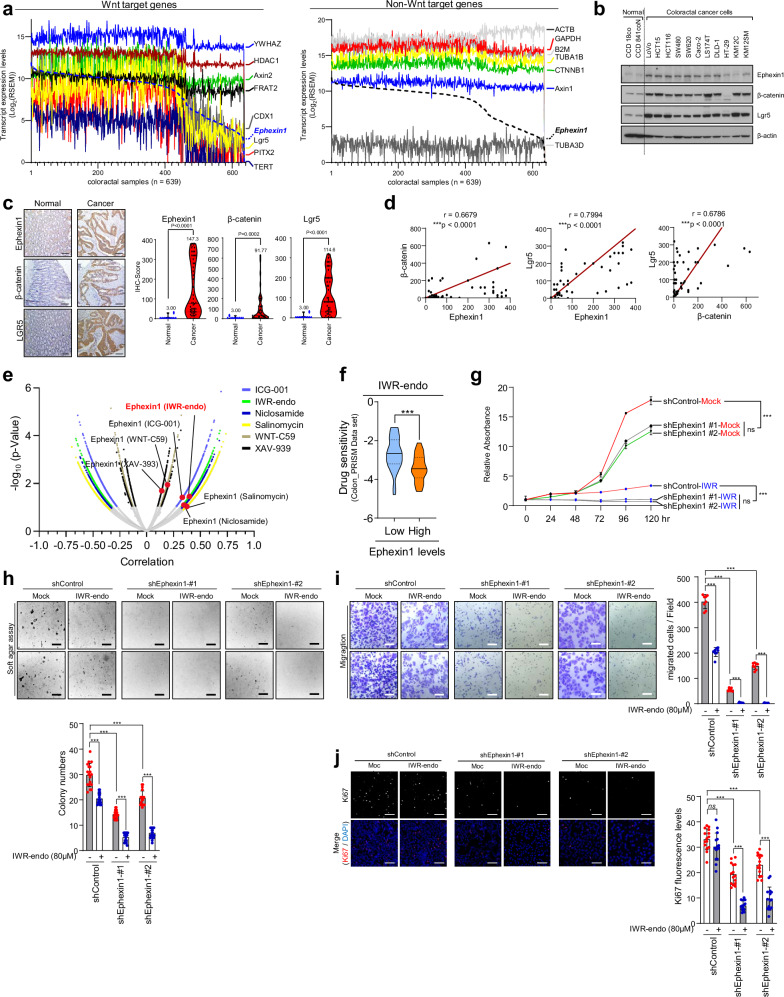
Ephexin1 and Wnt/β-catenin signaling are involved in colorectal cancer. **a** Analysis of Ephexin1 and Wnt/β-catenin target gene expression, along with Ephexin1 and non-Wnt target gene expression, in the TCGA-COAD and COADREAD cohorts (*n* = 639). **b** Western blot analysis of Ephexin1, β-catenin, and Lgr5 expression in normal and cancerous lung cell lines. **c** Immunohistochemical staining for Ephexin1, β-catenin, and Lgr5 in cancerous (*n* = 40) and corresponding normal tissues (*n* = 10). Scale bar = 100 μm. Immunohistochemistry (IHC) scores are presented as the means ± SEM, ***P < 0.0001 (Student’s *t*-test). **d** Correlations of IHC levels of Ephexin1 with β-catenin, Ephexin1 with Lgr5, and β-catenin with Lgr5. Correlated analyses are presented as the mean Spearman r, and *p* values are for a two-tailed Student’s *t*-test. ***p < 0.0001. **e** Volcano plot illustrating the relationship between sensitivity to Wnt/β-catenin inhibitors and Ephexin1 expression levels on the basis of RNAi screening data from DepMap. **f** Analysis of IWR1-endo drug (10 μM) sensitivity according to Ephexin1 mRNA expression levels in lung cancer cell lines (*n* = 60). **g** Effects of Ephexin1 knockdown on the proliferation of control and IWR1-endo (80 μM)-treated HCT116 cells. The data were presented as the means ± SEMs. ***p < 0.001, two-way ANOVA. **h**, **i** Effects of Ephexin1 knockdown on the anchorage-independent growth (**h**) and migration ability (**i**) of HCT116 cells, both untreated (control) and treated with IW;R1-endo (80 μM). Representative images are shown. Scale bar = 100 μm. The data were presented as the means ± SEMs. ***P < 0.0001, two-tailed Student’s *t*-test. **j** Effect of reducing Ephexin1 expression on the levels of the Ki67 proliferation marker in control and IWR1 endo-treated HCT116 cells. Representative images are shown. Scale bar = 100 μm. The data were presented as the means ± SEMs. ***P < 0.0001, two-tailed Student’s *t*-test.

To explore the potential link between Ephexin1 and Wnt/β-catenin signaling, we analyzed RNA sequencing data from the TCGA database, specifically the expression of Ephexin1 and β-catenin target genes in CRC samples (*n* = 639). Our analysis revealed a statistically significant positive correlation between Ephexin1 expression and the expression of Wnt/β-catenin target genes (Fig. 1a and Supplementary Fig. 1a, b). This correlation was specific to Wnt/β-catenin target genes, as it was not observed with non-Wnt/β-catenin target genes. These findings suggest that Ephexin1 selectively influences Wnt signaling in CRC. Furthermore, our research demonstrated that Ephexin1, along with β-catenin and Lgr5 (a key gene in the Wnt/β-catenin pathway), is significantly more highly expressed in CRC cell lines than in normal colon cells (Fig. 1b). Immunohistochemical analysis confirmed a positive correlation between the expression of Ephexin1, β-catenin, and Lgr5 in CRC tissues (Fig. 1c, d).

The uncontrolled proliferation of colorectal stem cells is a key mechanism in the development of CRC [36, 37]. Given the role of Lgr5 as a key marker for colorectal stem cells [38, 39],

we investigated the presence of Ephexin1 in Lgr5-positive stem cells specifically located in the crypt regions of the colon. Our results confirmed that Ephexin1 is uniquely expressed in these colonic stem cells (Supplementary Fig. 2a, b). Notably, both Ephexin1 and Lgr5 were highly expressed in the same regions within CRC tissues (Supplementary Fig. 2c). Moreover, exposure to Wnt3a-conditioned medium (Wnt3a-CM), which activates the Wnt/β-catenin signaling pathway, resulted in increased Ephexin1 expression at both the mRNA and protein levels (Supplementary Fig. 3a, b). These results indicate that Ephexin1 and Wnt/β-catenin signaling may be regulated by a positive feedback loop.

We further explored the relationship between Ephexin1 expression and sensitivity to drugs that inhibit Wnt/β-catenin. Analysis of data from the DepMap portal revealed that lower Ephexin1 levels were correlated with increased effectiveness of these inhibitors, suggesting a link between Ephexin1 expression and the efficacy of Wnt/β-catenin inhibitors (Fig. 1e, f and Supplementary Fig. 4). Compared with control treatment, treatment with the Wnt inhibitor IWR1-endo significantly reduced growth (Fig. 1g), anchorage-independent growth (Fig. 1h), and migration ability (Fig. 1i) in HCT116 cells deficient in Ephexin1. Additionally, cells with diminished Ephexin1 expression exhibited a notable decrease in the expression of the cell proliferation marker Ki67 following IWR1-endo treatment (Fig. 1j). These findings suggest that Ephexin1 plays a vital role in controlling Wnt/β-catenin signaling in CRC, underscoring its potential as a target for therapy.

### Ephexin1 influences the Wnt/β-catenin signaling pathway

**Fig. 2:**
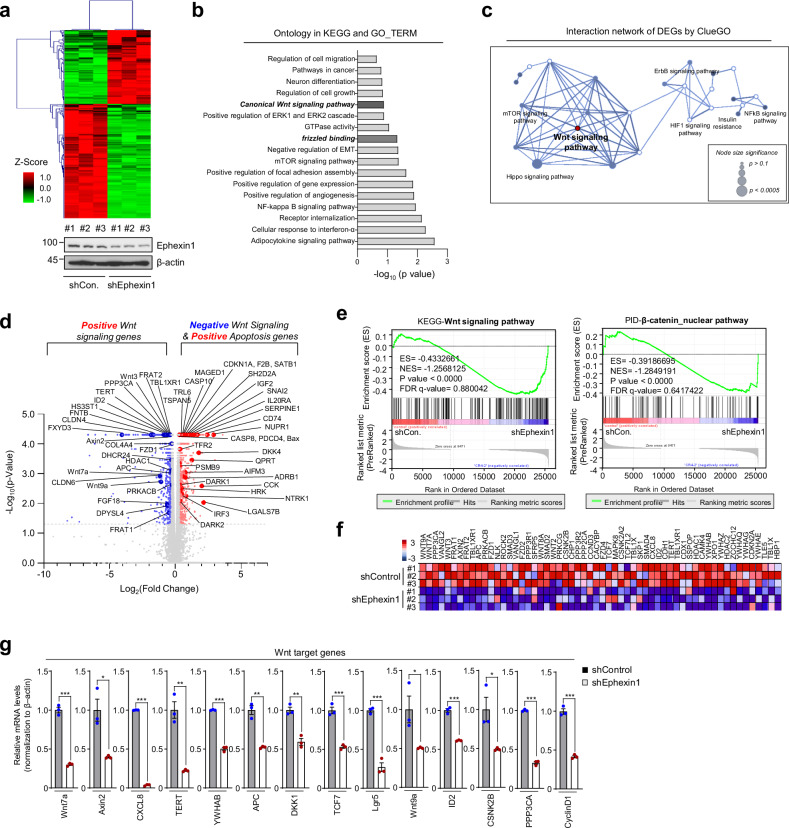
Ephexin1 enhances the Wnt/β-catenin signaling pathway and regulates Wnt/β-catenin target genes. **a** Heatmap showing the differentially expressed genes in control and Ephexin1-deficient HCT116 cells. Red and green indicate high and low mRNA expression levels, respectively. **b**, **c** RNA-seq datasets generated from control and Ephexin1-depleted HCT116 cells were analyzed to identify differentially expressed genes (DEGs), including both up- and downregulated genes. Gene ontology analysis of differentially expressed genes in KEGG and GO terms (**b**). Analysis of the interaction network of differentially expressed genes (DEGs) using ClueGO (https://apps.cytoscape.org/apps/cluego) (**c**). **d** Volcano plot showing the differentially expressed genes (with an FDR ≤0.05 and a fold change ≥2), including Wnt target genes, non-Wnt target genes, and apoptosis-related genes, in HCT116 cells depleted of Ephexin1 compared with control cells. Compared with the control, Ephexin1 knockdown resulted in the downregulation of 199 genes and the upregulation of 127 genes. The blue and red circles indicate the positions of these target genes. **e** Gene set enrichment analysis (GSEA) identified differentially expressed genes targeted by the Wnt signaling pathway and the β-catenin nuclear pathway between control and Ephexin1-depleted HCT116 cells. **f** Heatmap showing Wnt/β-catenin target genes downregulated and upregulated by Ephexin1 knockdown in HCT116 cells. **g** RT‒qPCR analysis comparing the expression of the specified Wnt target genes in control and Ephexin1-depleted HCT116 cells. The values represent the relative expression normalized to β-actin mRNA ± SEM. *p < 0.05; **p < 0.01; ***p < 0.001 compared with the control. *p* values are for a two-tailed Student’s *t*-test.

To investigate the role of Ephexin1 in the Wnt/β-catenin signaling pathway, we conducted RNA-Seq analysis on HCT116 cells with and without Ephexin1 knockdown. Our bioinformatics analysis identified 326 genes that were differentially expressed (Fig. 2a). We then focused on statistically significant gene changes using ontology and interaction network analysis of these differentially expressed genes (DEGs) along with functional enrichment analysis. These analyses demonstrated that the absence of Ephexin1 affects genes associated with Wnt signaling (Fig. 2b, c and Supplementary Fig. 5a). Specifically, genes normally activated by Wnt signaling were found to be suppressed in Ephexin1-knockdown HCT116 cells, whereas genes typically inhibited by Wnt signaling, along with genes activated by apoptosis, were increased (Fig. 2d). Furthermore, gene set enrichment analysis (GSEA) and heatmap data revealed a direct correlation between Ephexin1 levels and the activation of Wnt/β-catenin target genes (Fig. 2e, f). Through RT‒qPCR analysis, we confirmed significant differences in the expression of Wnt/β-catenin target genes in the Ephexin1-deficient cells compared with the control cells (Fig. 2g and Supplementary Fig. 5b). These results indicate that Ephexin1 plays a critical role in facilitating the positive regulation of genes downstream of the Wnt/β-catenin signaling pathway.

### Ephexin1 enhances the transcriptional activity of β-catenin in the Wnt signaling pathway

**Fig. 3:**
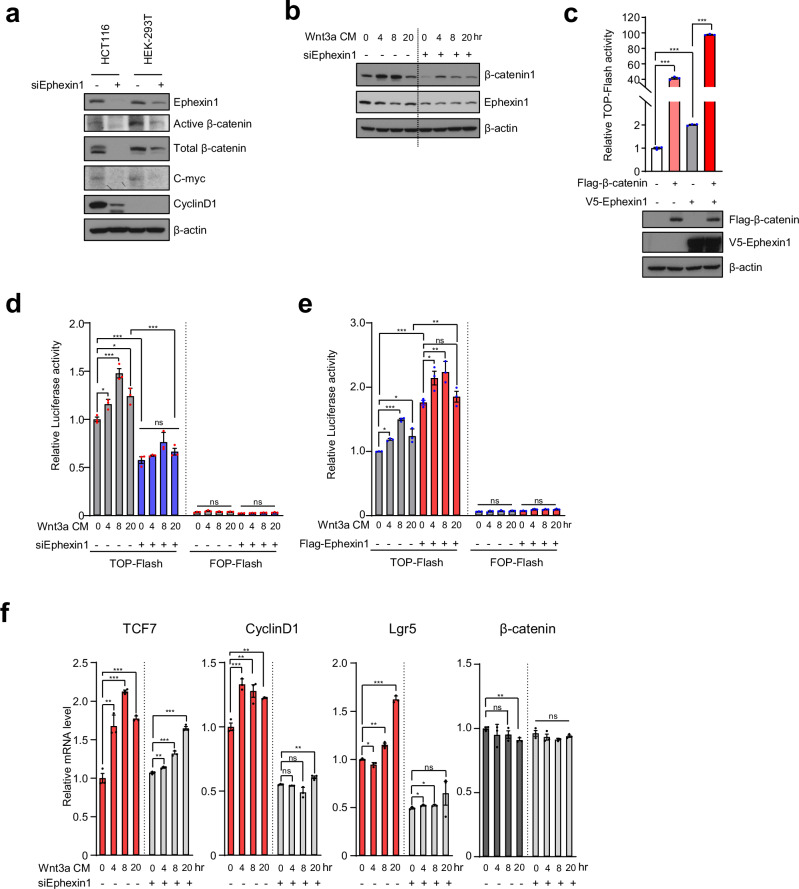
Ephexin1 increases the transcriptional activity of β-catenin. **a** HCT116 and HEK293T cells were transiently transfected with either control siRNA or Ephexin1 siRNAs. The cell lysates were then subjected to Western blot analysis with the indicated antibodies. **b** Control and Ephexin1-depleted HCT116 cells were treated with Wnt3a-CM for the indicated periods of time. The cell lysates were analyzed by Western blotting with the indicated antibodies. **c** TOP-Flash luciferase activity was analyzed in HCT116 cells after transfection with Flag-tagged β-catenin and/or V5-tagged Ephexin1. **d** The luciferase activities of TOP-Flash and FOP-Flash were measured in control and Ephexin1-depleted HCT116 cells after treatment with Wnt3a-CM. **e** The luciferase activities of TOP-Flash and FOP-Flash were measured in control and Flag-Ephexin1-overexpressing HCT116 cells following treatment with Wnt3a-CM. **f** Control and Ephexin1-depleted HCT116 cells were treated with Wnt3a-CM for the indicated periods of time, and the selected transcripts were analyzed via RT‒qPCR. The values represent the relative expression normalized to β-actin mRNA ± SEM. *p < 0.05; **p < 0.01; ***p < 0.001 compared with the control. *p* values are for a two-tailed Student’s *t-*test.

The primary role of the canonical Wnt/β-catenin signaling pathway is to regulate gene expression by allowing β-catenin to function as a transcription factor [40]. Thus, we hypothesized that Ephexin1 influences the transcriptional activity of β-catenin. We tested this hypothesis by reducing Ephexin1 in HCT116 and HEK293T cells. Phosphorylation of β-catenin leads to its degradation, whereas nonphosphorylation at these sites promotes stabilization [15, 41]. Our results revealed that Ephexin1 depletion decreased active β-catenin, total β-catenin, and the expression of its target genes, such as cyclin D1 and c-myc. Conversely, Ephexin1 overexpression increased β-catenin, cyclin D1, c-myc, and Lgr5 levels (Fig. 3a and Supplementary Fig. 6a, b). Moreover, Ephexin1 knockdown significantly reduced the Wnt3a-CM-mediated increase in β-catenin levels (Fig. 3b).

To further explore the effect of Ephexin1 on the transcriptional activity of β-catenin, we conducted a TOP-Flash luciferase reporter assay. In both HCT116 and HEK293T cells, simultaneous overexpression of Ephexin1 and β-catenin significantly enhanced the transcriptional activity of β-catenin more than did overexpression of either β-catenin or Ephexin1 alone (Fig. 3c and Supplementary Fig. 6c). Additionally, depleting Ephexin1 significantly reduced the TOP-Flash luciferase reporter activity induced by Wnt3a-CM (Fig. 3d and Supplementary Fig. 6d), whereas overexpressing Flag-tagged Ephexin1 markedly increased this activity (Fig. 3e and Supplementary Fig. 6e). Furthermore, treatment with Wnt3a-CM significantly increased the mRNA levels of TCF7, cyclin D1, and Lgr5, which are key target genes of the Wnt/β-catenin signaling pathway, in the control groups, but this effect was not observed in the Ephexin1-deficient HCT116 cells. On the other hand, the expression of the β-catenin gene, which is not directly targeted by Wnt/β-catenin signaling, remained unchanged (Fig. 3f). These findings collectively indicate that Ephexin1 plays a crucial role in enhancing the activity of β-catenin as a transcription factor.

### Ephexin1 interacts with the β-catenin destruction complex by binding to Axin1

**Fig. 4:**
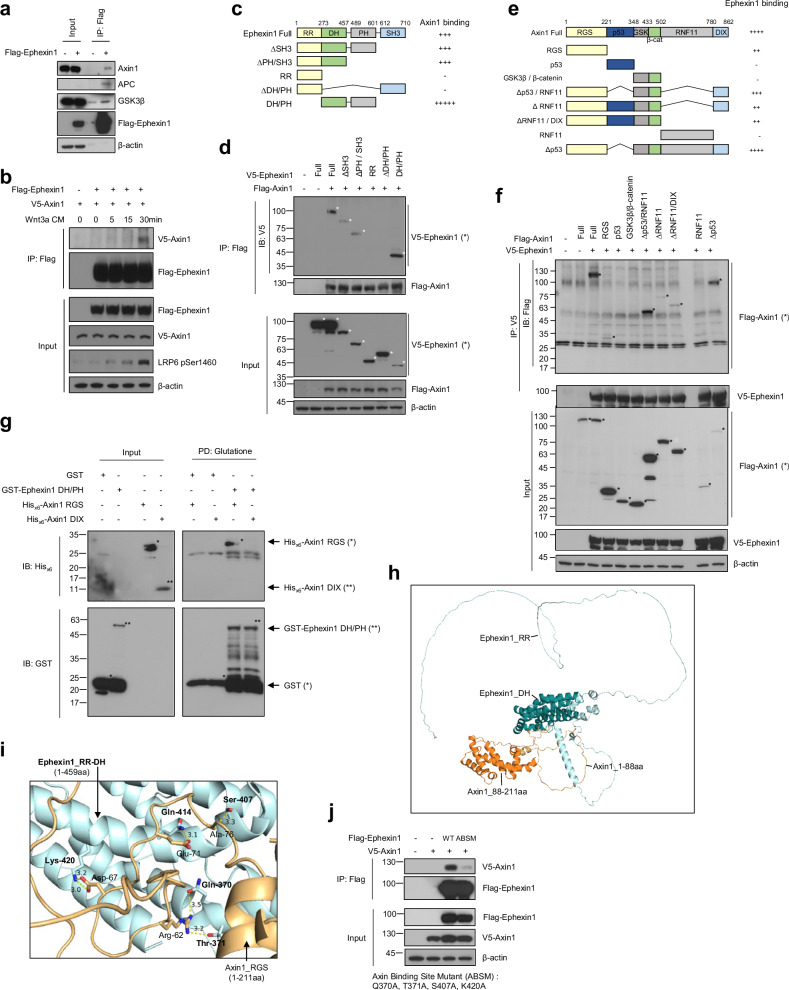
Ephexin1 interacts with the RGS of Axin1 via the DH domain. **a** HEK293T cells were immunoprecipitated with an anti-Flag antibody after transient transfection with Flag-tagged Ephexin1 and immunoblotted with the indicated antibodies. **b** Coimmunoprecipitation of protein extracts from HEK293T cells cotransfected with the proteins Ephexin1 (Flag-tagged) and Axin1 (V5-tagged) was performed with an anti-Flag antibody following treatment with Wnt3a-CM for the indicated durations. Western blot analysis was then performed with the indicated antibodies. **c** Schematic representation of the full-length and a series of deletion mutants of Ephexin1. A summary of the degree to which each protein interacts with Axin1 is shown on the right. **d** Protein extracts from HEK293T cells cotransfected with either V5-tagged full-length or mutant Ephexin1 along with Flag-tagged Axin1 were immunoprecipitated with an anti-Flag antibody and subjected to Western blot analysis with the indicated antibodies. **e** Schematic representation of full-length Axin1 and a series of Axin1 deletion mutants. A summary of the degree to which each protein interacts with Ephexin1 is shown to the right. **f** Lysates from HEK293T cells cotransfected with V5-tagged Ephexin1, and either full-length or mutant Flag-tagged Axin1 were subjected to immunoprecipitation with an anti-Flag antibody. Western blot analysis was then performed with V5 and Flag antibodies. **g** An in vitro GST pulldown assay was utilized to investigate the binding between the GST-DH/PH domain of Ephexin1 and either the recombinant Hisx6-tagged Axin1-RGS (1–221 aa) or the Axin1-DIX (780-862 aa) domain. GST alone was used as a negative control. **h** Using the AlphaFold-multimer, the most accurate predictions for the protein structures of Ephexin1_RR-DH (1–457 amino acids) in blue and Axin1_RGS (1–211 amino acids) in yellow were generated and subsequently visualized with PyMol software. **i** Hydrogen bonding sites within the predicted protein structures of Ephexin1_RR-DH (aa 1–457) and Axin1_RGS (aa 1–211) were analyzed using ChimeraX software (https://www.rbvi.ucsf.edu/chimerax/), and visualization was performed using PyMOL software (https://pymol.org/2/). **j** Coimmunoprecipitation analysis was conducted on protein extracts from HEK293T cells that were cotransfected with Flag-tagged Ephexin1 (either the wild-type or the ABSM mutant versions with the Q370A, T371A, S407A, and K420A mutations) and V5-tagged Axin1. Immunoprecipitation was performed using a Flag antibody, and the interaction between Ephexin1 and Axin1 was then assessed by Western blotting with an anti-V5 antibody.

Considering the role of Ephexin1 in regulating the transcriptional activity of β-catenin, we hypothesized that Ephexin1 potentially interacts with the β-catenin destruction complex. To test this hypothesis, we used both yeast two-hybrid assays and coimmunoprecipitation techniques. In the yeast two-hybrid assay, we discovered an interaction between Ephexin1 and Axin1, a key component of the β-catenin destruction complex (Supplementary Fig. 7a–c). This interaction was confirmed in HEK293T cells through coimmunoprecipitation, where Ephexin1 was found to bind not only with Axin1 but also with other elements of the destruction complex, such as β-catenin, TCF4, CK1, GSK3β, and APC (Fig. 4a and Supplementary Fig. 7d, e). Importantly, we observed that the interaction between Ephexin1 and Axin1 became stronger with increasing duration of Wnt3a CM treatment (Fig. 4b).

To identify which part of Ephexin1 interacts with Axin1, we examined five different Ephexin1 mutants, each lacking a specific domain of Ephexin1. Our results indicated that Axin1 attaches to the DH/PH domain of Ephexin1, which spans amino acids 273 to 601. In contrast, the RR and SH3 domains of Ephexin1 showed no affinity for binding to Axin1 (Fig. 4c, d). To further determine the specific domain of Axin1 involved in binding to Ephexin1, we generated and analyzed eight Flag-tagged Axin1 mutants. This analysis revealed that the RGS domain (aa 1–221) of Axin1 was crucial for binding to V5-tagged Ephexin1 (Fig. 4e, f). For direct binding confirmation between Ephexin1 and Axin1, we performed GST pulldown assays using recombinant proteins of GST-Ephexin1 (either full-length or containing the DH/PH domain) and His_x6_-Axin1 (RGS or DIX domain). GST pulldown assays confirmed a direct interaction between the DH/PH domain of Ephexin1 and the RGS domain of Axin1 without interacting with the DIX domain of Axin1 (Fig. 4g and Supplementary Fig. 8).

To further verify the interaction between Ephexin1 and Axin1, we used AlphaFold-multimer to predict how these proteins bind together [34, 42, 43], supporting our immunoprecipitation results. A precise analysis of the binding domain of Ephexin1 to Axin1 was necessary for accurate AlphaFold-multimer prediction, leading to the generation of seven Ephexin1 mutants with specific deletions in the DH/PH domain. We observed that the DH domain, but not the PH domain, of Ephexin1 is required for its interaction with Axin1 (Supplementary Fig. 9a, b).

To predict the stable structure of Ephexin1 accurately, we utilized the RR/DH domain (amino acids 1–457) of Ephexin1 and the RGS domain (amino acids 1–211) of Axin1 in AlphaFold-multimer modeling. This approach yielded predictions of a high-quality interaction between the RR/DH domain of Ephexin1 and the RGS domain of Axin1, as evidenced by sequence coverage, the predicted local distance difference test (pLDDT) confidence value, and the predicted alignment error (PAE) (Supplementary Fig. 10a–c). Further structural analysis indicated that the DH domain of Ephexin1 interacts with the RGS domain of Axin1 through the formation of four hydrogen bonds, underscoring the specificity and strength of this interaction (Fig. 4h, i). To further investigate this interaction, the four amino acids in the DH domain of Ephexin1 predicted to interact with Axin1 were replaced with alanine. The mutated Ephexin1 significantly reduced its interaction with Axin1 (Fig. 4j). Therefore, the hydrogen bonds between Ephexin1 and Axin1 are likely crucial for their interaction.

### Ephexin1 regulates β-catenin degradation through modulation of the Axin1 interaction network

**Fig. 5:**
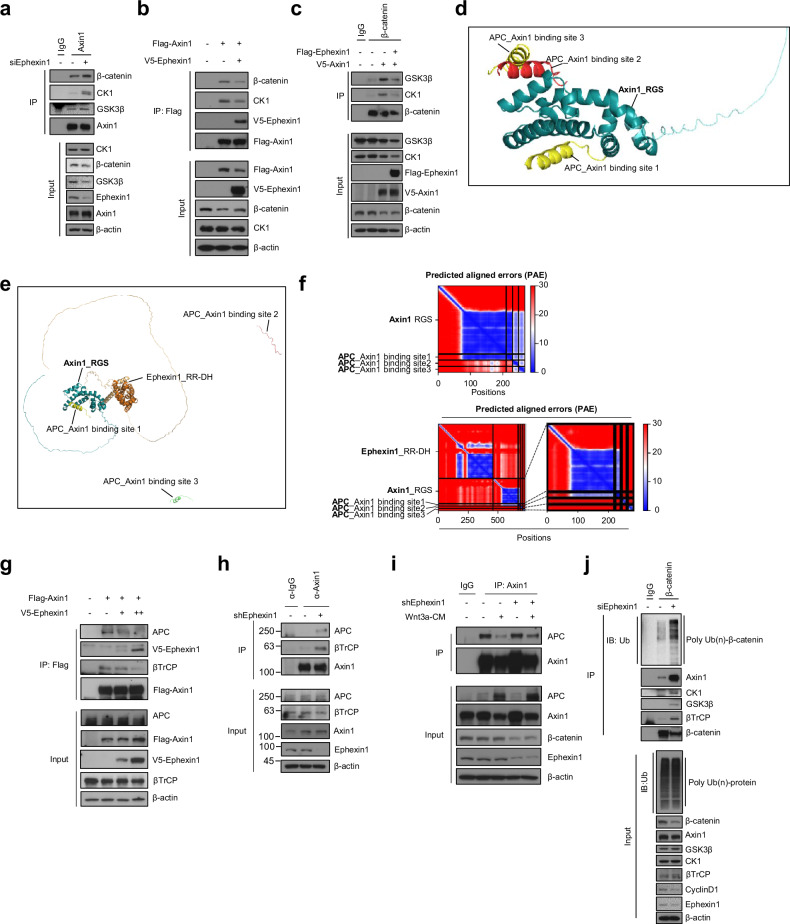
Interaction of Ephexin1 with Axin1 inhibits β-catenin ubiquitination. **a** Lysates from control and Ephexin1-depleted HEK293T cells were immunoprecipitated with an anti-Axin1 antibody and subjected to Western blot analysis with the indicated antibodies. **b** Lysates from HEK293T cells cotransfected with V5-tagged Ephexin1 and Flag-tagged Axin1 were immunoprecipitated with an anti-Axin1 antibody and subjected to Western blot analysis with the indicated antibodies. **c** Lysates from HEK293T cells, either transfected with V5-Axin1 alone or cotransfected with Flag-Ephexin1 and V5-Axin1, were immunoprecipitated with an anti-β-catenin antibody and subjected to Western blot analysis with the indicated antibodies. **d** Using AlphaFold-multimer, the best model for predicting the protein binding structure of the RGS domain of Axin1 (aa 1–211) in blue, along with the Axin1 binding sites 1, 2, and 3 (aa 1567–1595 in yellow, 1716–1734 in red, and 2032–2050 in green), was generated and subsequently visualized with PyMol software. **e** Using AlphaFold-multimer, the most accurate predictions for the protein binding structures of the RGS domain of Axin1 (aa 1–211) are shown in blue; Axin1 binding sites 1, 2, and 3 on APC (aa 1567–1595 in yellow, 1716–1734 in red, and 2032–2050 in green); and the RR-DH domain of Ephexin1 (aa 1–457 in orange), which were generated and subsequently visualized with PyMol software. **f** Comparison of the predicted alignment errors (PAEs) for the best models of the Axin1/APC complex and the Ephexin1/Axin1/APC complex, as determined by AlphaFold-multimer. **g** Lysates from HEK293T cells, either transfected with Flag-Axin1 alone or cotransfected with Flag-Axin1 and V5-Ephexin1, were immunoprecipitated with an anti-Flag antibody and subjected to Western blot analysis with the indicated antibodies. **h** Lysates from control and Ephexin1-depleted HCT116 cells were immunoprecipitated with an anti-Axin1 antibody and subjected to Western blot analysis with the indicated antibodies. **i** Control and Ephexin1-deficient HCT116 cells were treated with Wnt3a-CM or left untreated. The cell lysates were then immunoprecipitated with an anti-Axin1 antibody, followed by immunoblotting with the indicated antibodies. **j** Lysates from control and Ephexin1-depleted HCT116 cells were immunoprecipitated with an anti-β-catenin antibody and subjected to Western blot analysis with the indicated antibodies.

Considering the function of Axin1 in the Wnt signaling pathway as a scaffold protein that facilitates the proteasomal degradation of β-catenin [41], we hypothesized that the interaction of Ephexin1 with Axin1 could influence the degradation pathway of β-catenin via ubiquitination mechanisms. To investigate this hypothesis, we conducted immunoprecipitation experiments on Axin1 in HEK293T cells under conditions of both Ephexin1 deficiency and overexpression. Our findings indicated that the absence of Ephexin1 amplified the interactions between Axin1 and key proteins in the Wnt signaling pathway, such as β-catenin, CK1, and GSK3β (Fig. 5a), whereas overexpressing Ephexin1 diminished these interactions (Fig. 5b). The phosphorylation of β-catenin by CK1 and GSK3β is essential for its ubiquitination and subsequent degradation via the SCF^βTrCP^ complex [14, 15, 44, 45]. Interestingly, while overexpression of Axin1 increased the interaction of β-catenin with CK1 and GSK3β, this effect was mitigated by Ephexin1 overexpression (Fig. 5c and Supplementary Fig. 11).

Adenomatous polyposis coli (APC), a crucial scaffolding component, collaborates with Axin1 to promote the ubiquitination of β-catenin by the E3 ligase SCF^βTrCP^ [46]. Notably, the RGS domain of Axin1 attaches to three distinct regions on APC, at amino acids 1567–1595, 1716-1736, and 2032–2050 [47]. Our previous finding that Ephexin1 binds to the RGS domain of Axin1 (Fig. 4) suggests a competitive relationship between Ephexin1 and APC for the RGS domain of Axin1. To explore this hypothesis further, we used AlphaFold-multimer for predictive modeling. Our findings, in line with existing research, confirmed the interaction of Axin1 with APC through AlphaFold-multimer predictions (Fig. 5d and Supplementary Fig. 12a–c). Importantly, our predictive models indicated that the binding of Ephexin1 to Axin1 could interfere with the interaction between Axin1 and APC (Fig. 5e, f and Supplementary Fig. 12d–f). To validate these findings, we performed immunoprecipitation assays involving all three proteins. These experiments demonstrated that increasing Ephexin1 levels significantly decreased the interaction between Axin1 and APC, as well as between Axin1 and SCF^βTrCP^. Conversely, reducing Ephexin1 levels led to an increase in these interactions (Fig. 5g, h). Moreover, in HCT116 cells lacking Ephexin1, treatment with Wnt3a-CM caused a smaller reduction in the Axin1-APC interaction than in control cells, indicating that the absence of Ephexin1 allows for a more stable Axin1-APC connection (Fig. 5i). Additionally, analysis of β-catenin from Ephexin1-deficient HCT116 cells revealed increased interactions with components of the destruction complex and increased levels of β-catenin polyubiquitination (Fig. 5j). In contrast, overexpression of Ephexin1 decreased β-catenin polyubiquitination (Supplementary Fig. 13). These results indicate that Ephexin1 is essential for β-catenin stability, as it binds to Axin1, thereby inhibiting the activity of the β-catenin destruction complex and reducing β-catenin ubiquitination, which is crucial for its degradation.

### Ephexin1 depletion enhances the apoptotic and tumor-suppressive effects of Wnt inhibition in CRC

**Fig. 6:**
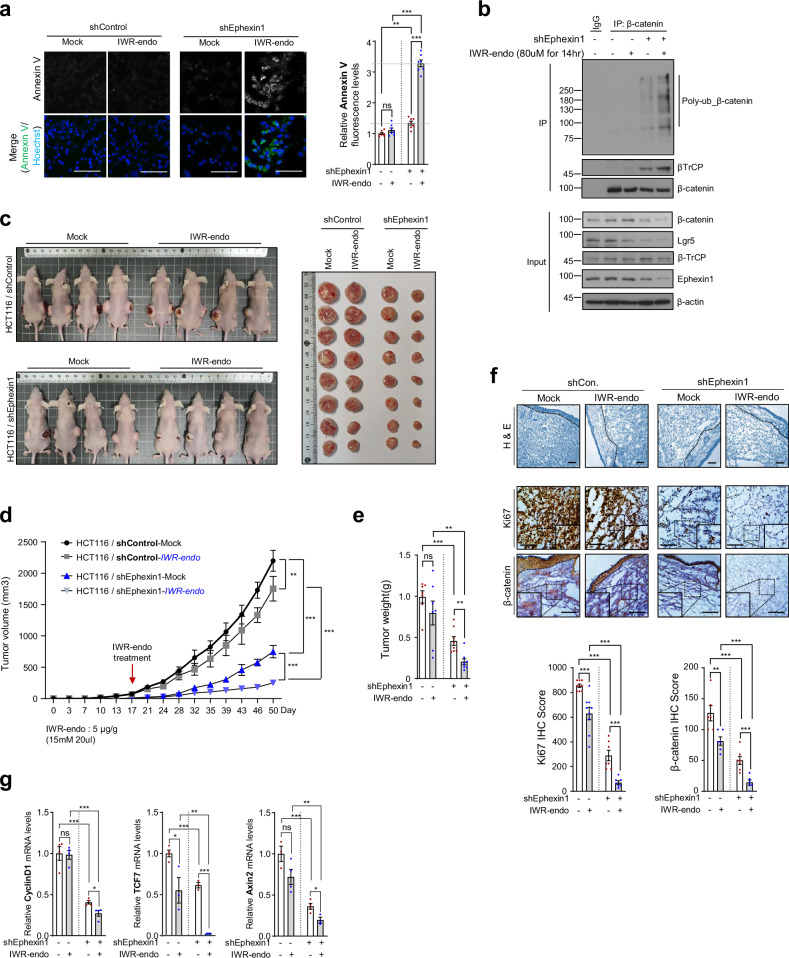
Ephexin1 deficiency enhances sensitivity to Wnt inhibitors. **a** Control and Ephexin1-deficient HCT116 cells were either treated with IWR1-endo (80 μM) or left untreated for 12 hours, followed by staining with Annexin V (green) for immunohistochemical analysis. The data were presented as the means ± SEMs. ns, not significant; **p < 0.01; ***p < 0.001, two-tailed Student’s *t*-test. **b** Control and Ephexin1-deficient HCT116 cells were treated with Wnt3a-CM or left untreated. The cell lysates were then immunoprecipitated with an anti-β-catenin antibody, followed by immunoblotting with the indicated antibodies. **c** Control and Ephexin1-depleted HCT116 cells were subcutaneously inoculated into BALB/c nude mice (*n* = 4). IWR1-endo (5 mg/kg) was administered every 3 or 4 days starting 17 days after transplantation. **d** The recorded tumor volumes for each group at the indicated times are shown. The data were presented as the means ± SEMs. ns, not significant; ***p < 0.001, two-way ANOVA. **e** The average tumor weight for each group at the endpoint of the experiment is presented. The data were presented as the means ± SEMs. ns, not significant; **p < 0.01; ***p < 0.001, two-tailed Student’s *t*-test. **f** H&E staining and immunohistochemistry analysis of Ki67 and β-catenin in each group at the endpoint of the experiment are shown. Scale bar = 100 μm. Quantification of Ki67 and β-catenin staining in each group is shown. The data were presented as the means ± SEMs. **p < 0.01; ***p < 0.001, two-tailed Student’s *t*-test. **g** The mRNA levels of Wnt/β-catenin target genes in the HCT116 xenograft tumors described in (**c**) were analyzed by qRT-PCR.

To explore the therapeutic potential of Ephexin1 in CRC, we evaluated the impact of Ephexin1 depletion on the effectiveness of a Wnt inhibitor. Initially, we assessed apoptotic responses in various CRC cell lines treated with IWR-endo and discovered that most cells underwent apoptosis following IWR1-endo treatment. However, HCT116 cells did not undergo apoptosis following IWR1-endo treatment (Supplementary Fig. 14a), indicating resistance to this treatment. Further investigation of the effects of IWR1-endo on both control and Ephexin1-depleted HCT116 cells, followed by apoptosis measurement through Annexin V staining, revealed that Ephexin1 depletion significantly increased apoptosis in HCT116 cells treated with IWR1-endo (Fig. 6a). To determine whether these effects were related to the Wnt signaling pathway, we analyzed β-catenin stability and target gene expression in both control and Ephexin1-depleted HCT116 cells. Compared with control cells, Ephexin1-depleted HCT116 cells subjected to IWR1-endo treatment exhibited a more pronounced decrease in the expression of β-catenin and its target gene cyclin D1 (Supplementary Fig. 14b, c). In support of this observation, treatment of Ephexin1-depleted HCT116 cells with IWR1-endo further increased β-catenin ubiquitination (Fig. 6b).

Next, to assess the ability of Ephexin1 to counteract Wnt inhibitor resistance in xenograft models, we administered IWR1-Endo to mice with tumors derived from either control HCT116 cells or Ephexin1-deficient HCT116 cells. The treatment was applied once every 3 to 4 days for a total of 33 days. While a slight reduction in tumor volume was observed in the control group, tumors derived from Ephexin1-deficient HCT116 cells exhibited a significant decrease in both volume and weight following IWR1-Endo treatment (Fig. 6c–e). Immunohistochemical analysis of tumor tissues further confirmed that depletion of Ephexin1 combined with IWR1-endo treatment led to reduced levels of Ki67 and β-catenin (Fig. 6f) and decreased expression of Wnt/β-catenin target genes and Wnt ligand genes (Fig. 6g and Supplementary Fig. 14d) compared with those in the controls. These findings indicate that the absence of Ephexin1 enhances the tumor-suppressive effects of Wnt pathway inhibitors in CRC, suggesting a promising strategy for tumor suppression.

### The interaction of Ephexin1 and Axin1 has clinical relevance in CRC

**Fig. 7:**
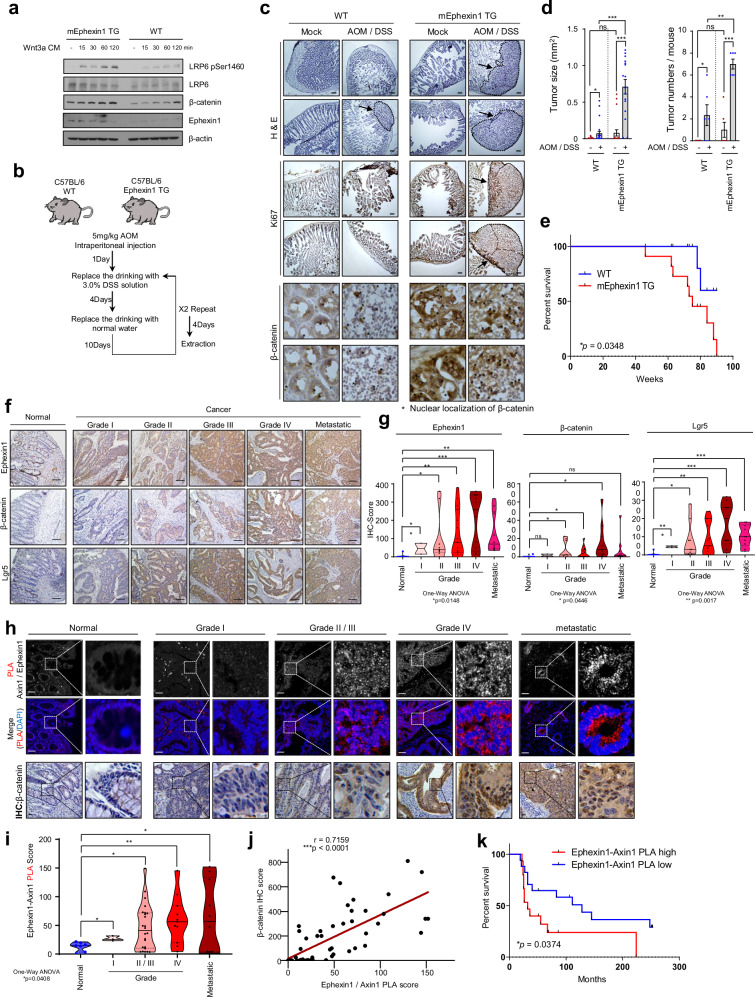
Interaction between Ephexin1 and Axin1 is associated with poor prognosis in CRC patients. **a** Western blot analysis was conducted to observe the time-dependent effects of Wnt3a-CM treatment on MEFs from WT (wild-type) and mEphexin1 TG (transgenic) mice. **b** Experimental schedule for establishing a colorectal cancer animal model using the AOM/DSS method in wild-type (WT) and Ephexin1 transgenic (TG) mice. **c** H&E and Ki67 staining of colorectal tissue from WT and mEphexin1 TG mice after induction of inflammatory CRC by AOM/DSS for 2 weeks. Arrows indicate areas of CRC. Scale bar = 500 μm. **d** The average tumor size and number of tumors in each group are presented. The data were presented as the means ± SEMs. ns, not significant; *p < 0.05; ***p < 0.001, two-tailed Student’s t-test. **e** Kaplan–Meier survival curves are shown for WT and mEphexin1 TG mice (*n* = 11). *p* values are from a log-rank test. **f** Immunohistochemical staining was used to evaluate Ephexin1, β-catenin, and Lgr5 expression in normal, Grade I/II, Grade III/IV, and metastatic CRC tissues, along with their corresponding normal tissues. Hematoxylin was used as the counterstain. Scale bar = 100 μm. **g** Expression scores for Ephexin1, β-catenin, and Lgr5 in a CRC tissue microarray containing 9 normal mucosa tissue samples, 2 Grade I samples, 9 Grade II samples, 15 Grade III samples, 12 Grade IV samples, and 10 metastatic tumor tissue samples. The data are presented as the means ± SEMs. *p* values are from the Mann–Whitney test. ns, not significant; *p < 0.05; **p < 0.01; ***p < 0.001. **h** A proximity ligation assay (PLA) was performed to identify interactions between Ephexin1 and Axin1 (red) in Grade I (*n* = 2), Grade II/III (*n* = 24), Grade IV (*n* = 12), and metastatic (*n* = 10) colorectal cancer tissues and their corresponding normal tissues (*n* = 9). DAPI (blue) was used as the counterstain to visualize the nuclei. Scale bar = 100 μm. **i** Quantification of the PLA data shown in (**h**). The data were presented as the means ± SEMs. *p* values are from a Mann–Whitney test. ns, not significant; *p < 0.05; **p < 0.01. **j** Correlation analysis between PLA (Ephexin1-Axin1) and β-catenin expression in CRC tissue. Correlated analyses are presented as the mean Spearman r, and *p* values are for a two-tailed Student’s *t-*test. ***p < 0.0001. **k** Kaplan–Meier graph representing overall survival rates for patients with the interaction between Ephexin1 and Axin1 in CRC. High Ephexin1-Axin1 PLA, *n* = 23; low Ephexin1-Axin1 PLA, *n* = 18. *p* values are from a log-rank test.

To investigate the role of Ephexin1 overexpression in CRC development, we generated transgenic (TG) mice that overexpress the mouse Ephexin1 (mEphexin1) gene (Supplementary Fig. 15). Compared with control treatment, treatment with Wnt3a-CM significantly increased LRP6 phosphorylation and β-catenin accumulation in mouse embryonic fibroblast (MEF) cells derived from these genetically modified mice (Fig. 7a). Given the established role of azoxymethane (AOM) and dextran sodium sulfate (DSS) in inducing CRC through Wnt/β-catenin signaling activation [48–50], we initiated colon cancer in both wild-type and mEphexin1 TG mice using AOM/DSS treatment over 2 weeks. Compared with control mice, mEphexin1 TG mice exhibited increased sizes and numbers of colon tumors. Furthermore, both Ki67 and β-catenin levels were elevated in the colon tissues of mEphexin1 TG mice, with a notable increase in nuclear β-catenin localization (Fig. 7b–d). A long-term survival analysis revealed that mEphexin1 overexpression significantly decreased the survival rate of the mice (Fig. 7e).

To assess the clinical relevance of Ephexin1 alongside β-catenin and Axin1 in human CRC, we examined tissue microarrays of colorectal tissues, including normal tissues, carcinomas of varying grades, and metastatic tumors. The expression levels of Ephexin1, β-catenin, and Axin1 were markedly greater in CRC tissues than in normal tissues. Notably, these levels increased progressively with increasing tumor grade and the presence of metastatic tumors. Patients with high levels of Ephexin1 expression had a poor prognosis. (Fig. 7f, g and Supplementary Fig. 16a). A proximity ligation assay (PLA) was used to examine the interactions between Ephexin1 and Axin1 and the quantitative changes in β-catenin in CRC patient samples. PLA foci indicating Ephexin1 and Axin1 interactions were markedly greater in cancer tissues than in normal tissues and significantly increased with tumor grade and metastasis (Fig. 7h, i). Notably, the PLA foci scores for Ephexin1 and Axin1 were positively correlated with the β-catenin immunohistochemistry scores. The group with higher Ephexin1-Axin1 binding levels had a more advanced tumor grade distribution and a poorer prognosis (Fig. 7j, k and Supplementary Fig. 16b). These findings emphasize the importance of the interaction between Ephexin1 and Axin1, suggesting that inhibiting this interaction could be a promising strategy for the development of anticancer drugs targeting Wnt/β-catenin in active CRC.

## Discussion

The Wnt/β-catenin signaling pathway is crucial in cancer development and progression, making it a key subject of research in CRC. Our study revealed that Ephexin1, which is significantly overexpressed in CRC, activates the Wnt/β-catenin signaling pathway. Furthermore, we highlight the critical role of Ephexin1 in promoting tumor growth through the activation of the Wnt/β-catenin pathway. This discovery enhances our understanding of the mechanisms underlying Wnt signaling dysregulation, which plays a crucial role in the progression of CRC, and introduces new possibilities for therapeutic interventions.

Ephexin1, which is traditionally known for its role in neurophysiological processes and minimal expression outside the nervous system [19, 22, 24], has emerged as a pivotal player in CRC through its overexpression and interaction with the Wnt/β-catenin signaling pathway. Our findings, corroborated by TCGA data analysis, establish a significant correlation between Ephexin1 expression and the activation of Wnt/β-catenin target genes in CRC. This relationship underscores the contribution of Ephexin1 to the hyperactive Wnt signaling observed in CRC, which drives the uncontrolled proliferation of colorectal stem cells and tumor growth. The unique impact of Ephexin1 on Wnt/β-catenin target genes, which was not observed in the nontarget genes of the Wnt/β-catenin signaling pathway, underscores its specific role in regulating this signaling pathway in CRC. This effect was further substantiated by our immunohistochemical analyses and RNA sequencing data, which revealed greater expression of Ephexin1, β-catenin, and Lgr5 in CRC cell lines and tissues than in normal colon cells. These observations suggest that Ephexin1 not only facilitates Wnt/β-catenin signaling but also enhances the transcriptional activity of β-catenin, thus promoting oncogenic gene expression.

The canonical Wnt/β-catenin pathway, a key regulator of cellular proliferation and differentiation, is frequently dysregulated in various cancers, including CRC [5–7]. Our study explored the underlying mechanisms of Ephexin1 by demonstrating its interaction with Axin1, a key component of the β-catenin destruction complex. This interaction plays a vital role in controlling the stability of β-catenin and, consequently, the activity of the Wnt/β-catenin signaling pathway. The binding of Ephexin1 to Axin1, particularly when Wnt signaling is increased, indicates a novel regulatory mechanism. This mechanism suggests that Ephexin1 could prevent the degradation of β-catenin, thus maintaining its oncogenic potential. The specificity of this interaction, delineated through various experimental approaches, including yeast two-hybrid assays, coimmunoprecipitation, GST pulldown assays, and structural predictions based on AlphaFold2 models, highlights the potential of targeting the Ephexin1-Axin1 interaction as a therapeutic strategy. Given the oncogenic nature of Ephexin1 and the tumor-suppressive function of Axin1, the overexpression of Ephexin1 in CRC suggests that it could counteract the effect of Axin1, thereby promoting cancer progression.

The finding that CRC cells lacking Ephexin1 exhibit increased sensitivity to inhibitors of the Wnt/β-catenin pathway highlights the potential benefits of targeting Ephexin1 for therapeutic purposes. By exploiting this sensitivity, the resistance faced by treatments targeting the Wnt pathway can potentially be overcome, offering a new approach to CRC treatment. Moreover, developing treatments that interfere with the interaction between Ephexin1 and Axin1 could provide an advanced way to control the stability of β-catenin and the activity of Wnt signaling in CRC. Our findings also emphasize the clinical importance of Ephexin1 and its interaction with Axin1 in CRC progression. The association between Ephexin1-Axin1 binding and poor patient outcomes, as evidenced by tissue microarray analysis and proximity ligation assays, highlights the critical need for further research into targeting this interaction as a therapeutic strategy. Disruption of this interaction could restore the β-catenin degradation pathway, consequently reducing Wnt signaling and its cancer-promoting role in CRC. Therefore, Ephexin1 is emerging as a potential biomarker for tracking cancer progression and an attractive target for developing new therapies specific to CRC. While clinical trials have been used to evaluate Wnt-targeted inhibitors such as vantictumab, ipafricept, and cetuximab [51–53], none have yet received FDA approval. The difficulty in creating effective therapies that target the Wnt/β-catenin pathway could be due to its essential role in maintaining tissue homeostasis [54]. The reported side effects of these therapies, such as bone toxicity, hair loss, skin rashes, and gastrointestinal issues [55–58], emphasize the necessity of targeting Wnt/β-catenin signaling in a more specific and safer manner. Focusing on anticancer drugs that target Ephexin1, which is mainly found in brain tissue but significantly increased in LC and CRC [19, 20, 22, 24], could offer specificity and effectiveness against cancer cells with minimal impact on normal cells. Given the complexity of the Wnt pathway, with its numerous components and regulatory mechanisms, future research should focus on ensuring that targeting Ephexin1 does not disrupt normal cell functions or produce adverse effects.

Intestinal cells renew every 5 days through the Wnt/β-catenin signaling pathway, which is crucial for tissue equilibrium and stem cell regeneration in the intestinal crypts [2, 3, 59]. These stem cells facilitate surface growth, but their excessive proliferation may lead to CRC [60, 61]. Approximately 80% of CRCs result from mutations in Wnt/β-catenin signaling genes [62–64], and ~37% involve K-Ras oncogene mutations [65, 66]. These mutations cause deregulation of the Wnt and Ras pathways, leading to uncontrolled stem cell growth and cancer [2, 67, 68]. Our findings indicate that Ephexin1, which is markedly increased in cancer stem cells from CRC patients, is influenced by K-Ras mutations [22, 24] and impacts the Wnt/β-catenin pathway, potentially leading to unregulated stem cell proliferation. Our future research will continue to further explore this effect.

In summary, this study elucidates the pivotal role of Ephexin1 in CRC by demonstrating its overexpression and direct influence on the Wnt/β-catenin signaling pathway. The interaction of Ephexin1 with Axin1 modulates the stability of β-catenin, suggesting a novel mechanism for Wnt pathway dysregulation in CRC (Supplementary Fig. 17). The development of drugs that specifically inhibit Ephexin1 or its interaction with Axin1 represents a new strategy for CRC therapy, particularly for Wnt-driven CRC. This approach, potentially in combination with existing Wnt pathway inhibitors, could improve treatment outcomes.

## Supplementary information

Supplementary information

## Data Availability

The RNA-seq data were deposited in the NCBI Gene Expression Omnibus (GEO) and are accessible through GEO series accession number GSE220669.
