# Supplementary material for: Disruption of the β-catenin destruction complex via Ephexin1-Axin1 interaction promotes colorectal cancer proliferation
Source: Exp Mol Med. 2025 Jan 1;57(1):151–66. doi: 10.1038/s12276-024-01381-1 (PMC11799323; doi:10.1038/s12276-024-01381-1)
Supplement: Supplementary file 1 — Supplementary information [file 12276_2024_1381_MOESM1_ESM.pdf]

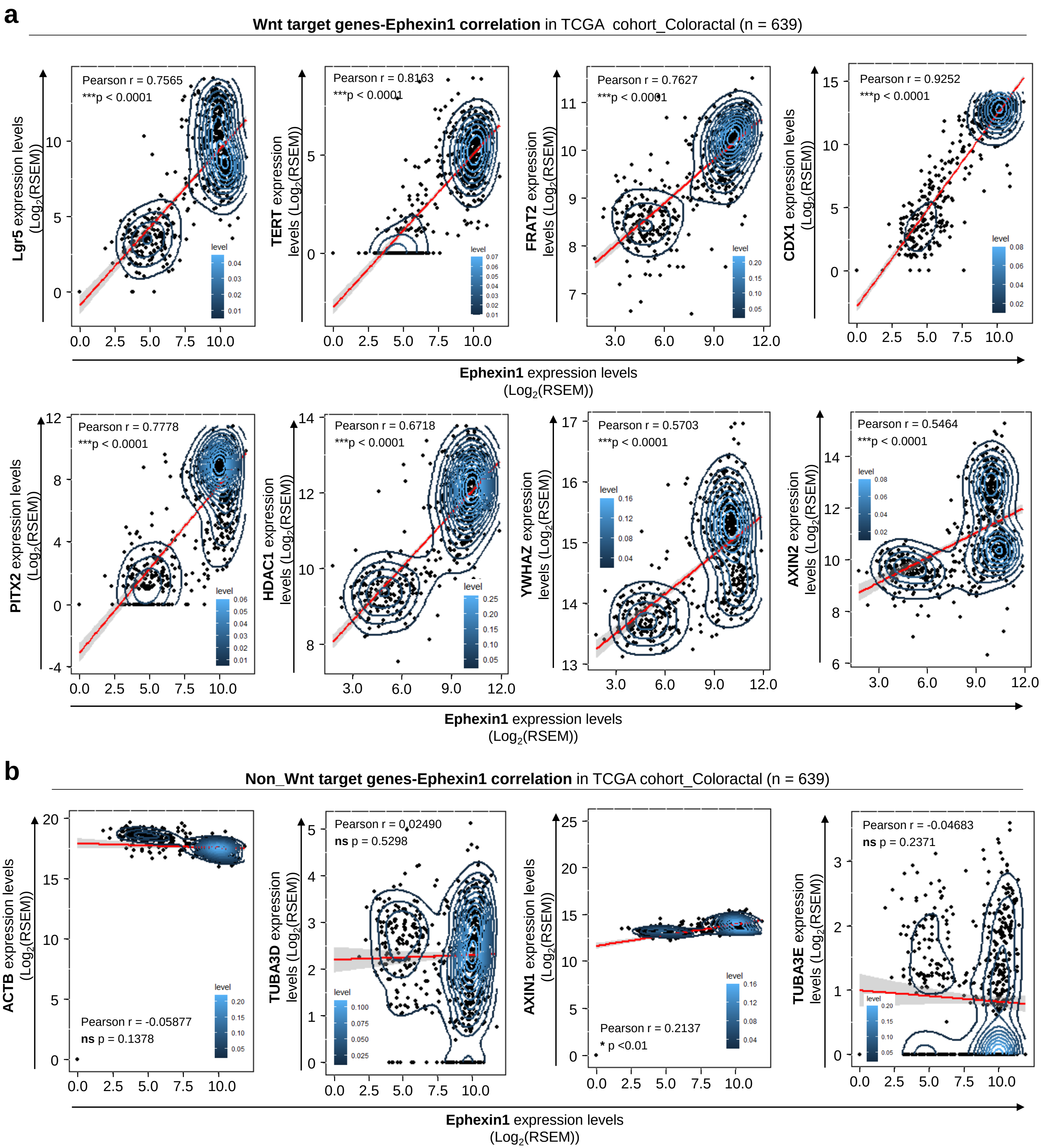

Supplementary Fig. 1 Correlation analysis between Ephexin1 expression and Wnt target genes using bioinformatics analysis in TCGA cohort. a, b Correlation analysis between Ephexin1 and Wnt/β-catenin target genes (**a**) or non-Wnt/β-catenin target genes (**b**) in the TCGA-COAD and COADREAD cohorts, respectively. Statistical processing and visualization were performed using the R program. A narrower contour indicates a higher density of data points. The red line illustrates the correlation, while the shaded area represents the regression analysis. The Pearson correlation coefficient method was employed for the correlation analysis.

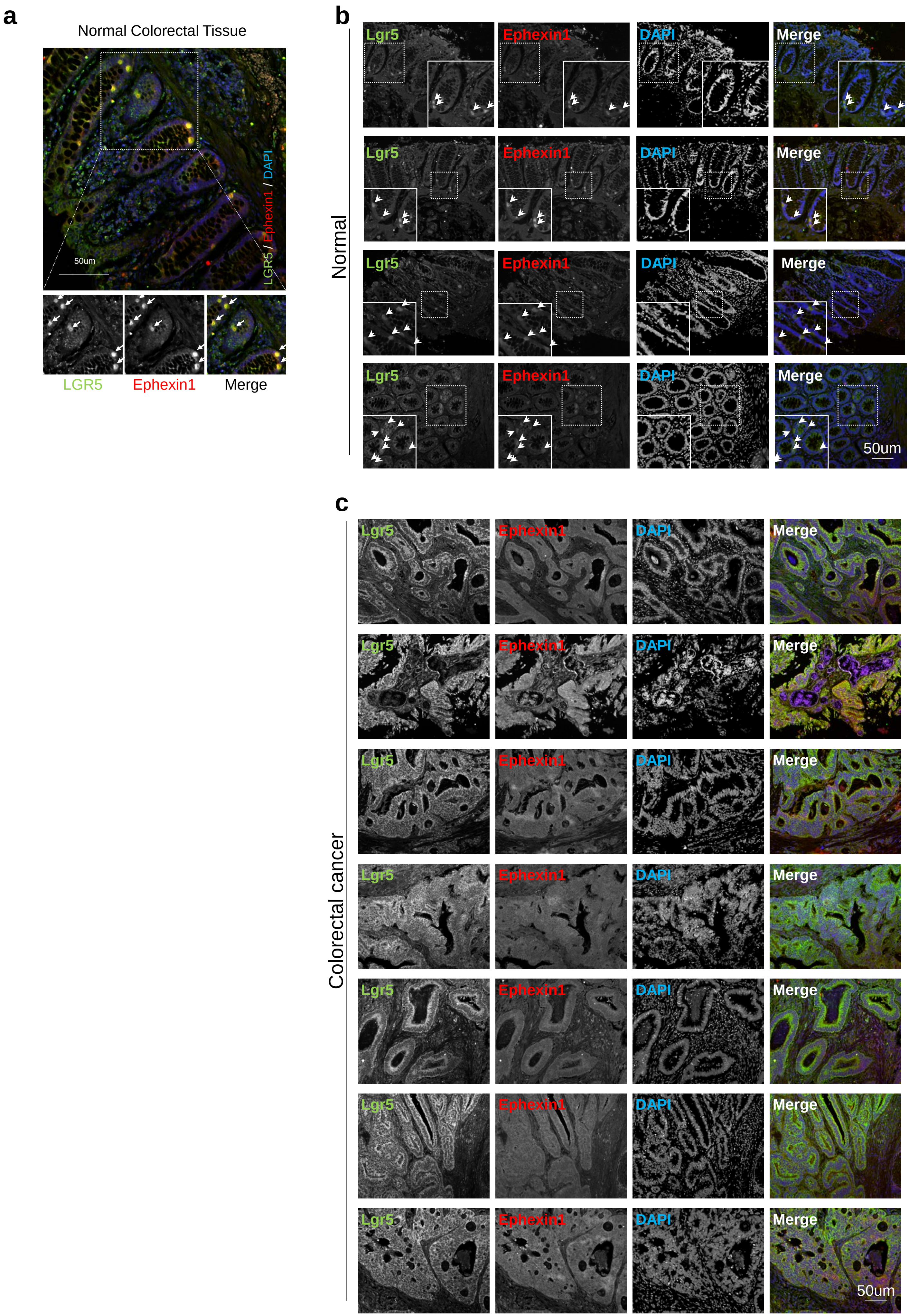
a
Normal Colorectal Tissue
LGR5 / Ephexin1 / DAPI
50um
LGR5
Ephexin1
Merge
b
Normal
Lgr5
Ephexin1
DAPI
Merge
50um
c
Colorectal cancer
Lgr5
Ephexin1
DAPI
Merge
50um

Supplementary Fig. 2. Comparison of the expression levels of Lgr5 and Ephexin1 in colorectal tissue using fluorescence Staining. a Representative images of fluorescence staining for Lgr5 and Ephexin1 in normal colon tissue. Scale bar = 50 μm. **b, c** Fluorescence staining was used to analyze the expression of Lgr5 (green) and Ephexin1 (red) in both normal colon tissue (**b**) and colon cancer tissue (**c**). DAPI (blue) stains the nuclei and serves as a counterstain. Arrows indicate Lgr5-positive cells and cells co-expressing Ephexin1. Scale bar = 50 μm.

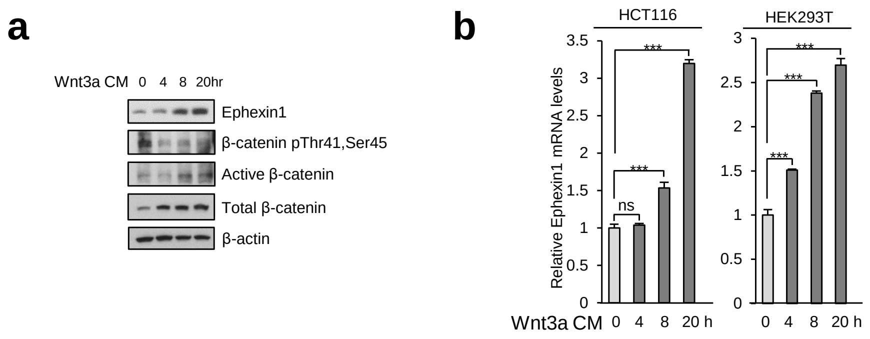

Supplementary Fig. 3 Wnt3a-CM treatment increases the expression of Ephexin1. a HCT116 cells were treated with Wnt3a-CM for the indicated time periods. Cell lysates were then subjected to Western blot analysis with the indicated antibodies. **b** HCT116 and HEK293T cells were treated with Wnt3a-CM for the indicated periods of time, after which select transcripts were evaluated using RT-qPCR. The values denote relative expression normalized to β-actin mRNA ± SEM. ns, not significant; *$p < 0.05$; **$p < 0.01$; ***$p < 0.001$ compared with control. p values are for a two-tailed Student's t-test.

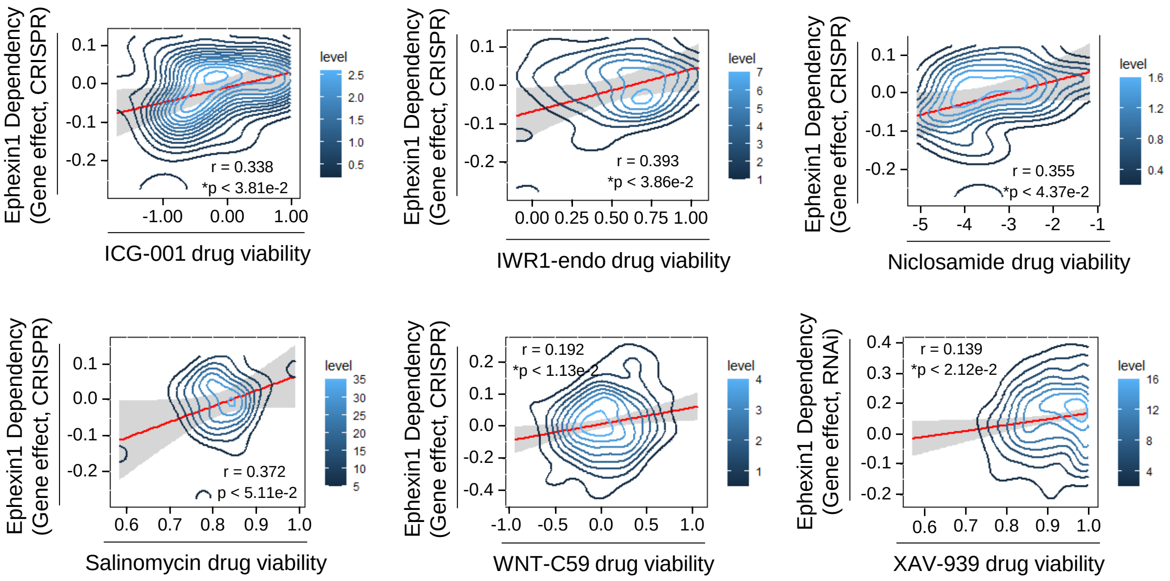

Supplementary Fig. 4 Positive correlation between Wnt/β-catenin targeted drug sensitivity and Ephexin1 expression. The R program was utilized for statistical processing and visualization. A narrower contour indicates a higher data distribution. The red straight line illustrates the correlation, while the gray area represents the regression analysis. The Pearson correlation coefficient method was utilized for the correlation analysis.

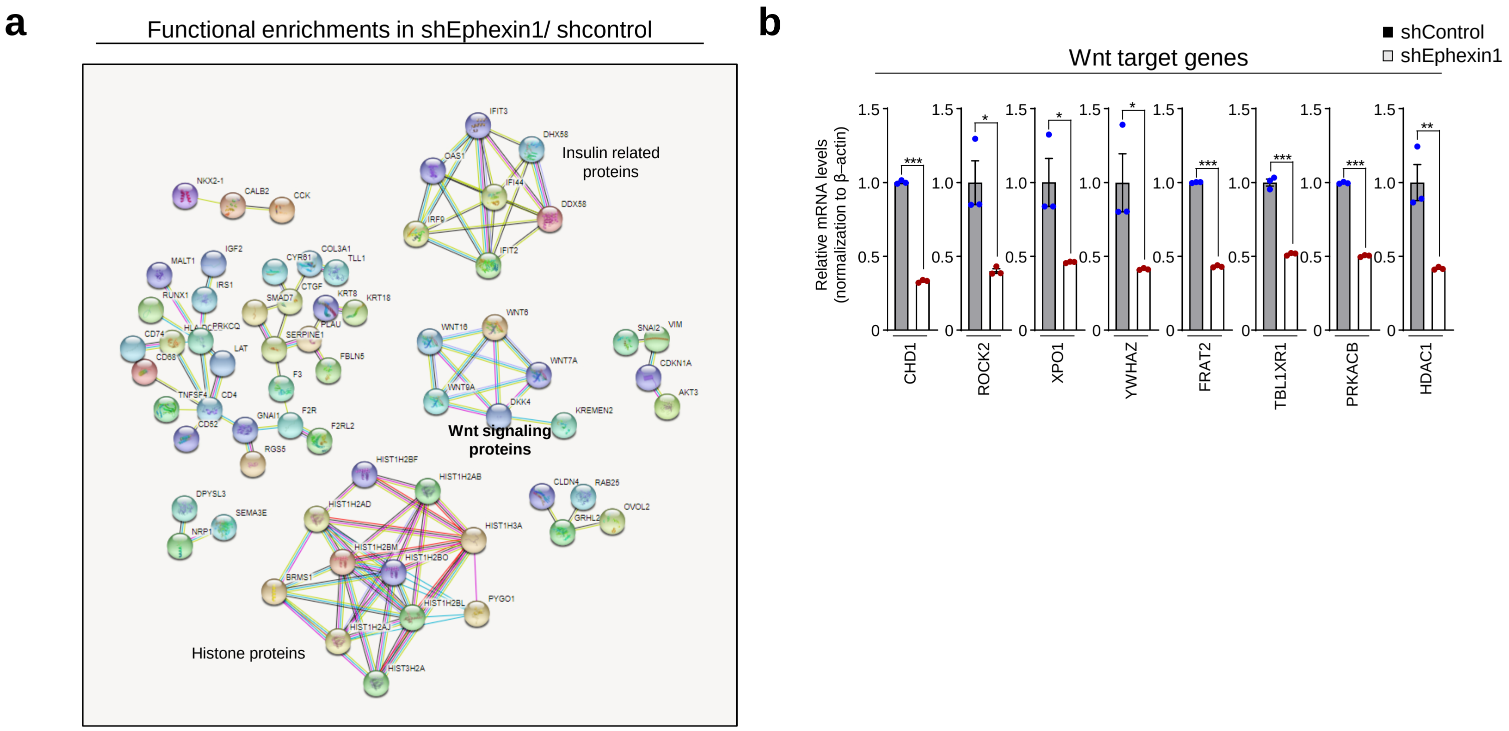

Supplementary Fig. 5 Functional enrichment analysis and transcript analysis of indicated genes in Ephexin1-depleted HCT116 cells. a The functional enrichment analysis illustrated the biological pathways and processes associated with correlated genes. This analysis was conducted using ClueGO software (https://apps.cytoscape.org/apps/cluego), and visualization was facilitated by Cytoscape 3.10.1. **b** RT-qPCR analysis was carried out on the indicated genes in Ephexin1-depleted HCT116 cells. Values represent the relative expression normalized to β-actin mRNA ± SEM. *$P < 0.05$; **$P < 0.01$; ***$P < 0.001$ compared with control. P values are for a two-tailed Student's *t*-test.

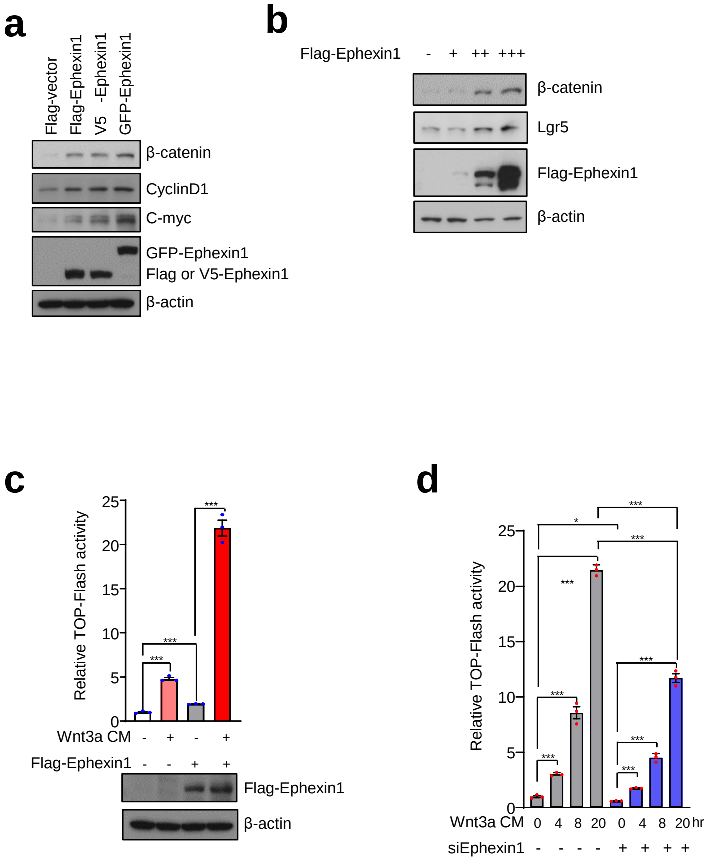

Supplementary Fig. 6 Ephexin1 regulates the transcriptional role of b-catenin. a HEK293T cells were transfected with three differently labeled Ephexin1 constructs. Cell lysates were analyzed by Western blot using the indicated antibodies. **b** The levels of β-catenin, Lgr5, and Ephexin1 proteins in HEK293T cells transfected with either a control vector or increasing amounts of Flag-tagged Ephexin1 were assessed. Cell lysates were subjected to Western blot analysis with the indicated antibodies. **c** TOP-Flash luciferase activity was measured in HEK293T cells after the transfection of Flag-tagged β-catenin and/or treatment with Wnt3a-CM. **d** TOP-Flash luciferase activity was analyzed after treatment with Wnt3a-CM in control or Ephexin1-depleted HEK293T cells. **e** The luciferase activity assay was conducted after transfection with Flag-tagged Ephexin1 and/or treatment with Wnt3a-CM in HEK293T cells. *P < 0.05; **P < 0.01; ***P < 0.001 compared with control. P values are for a two-tailed Student's *t*-test.

a

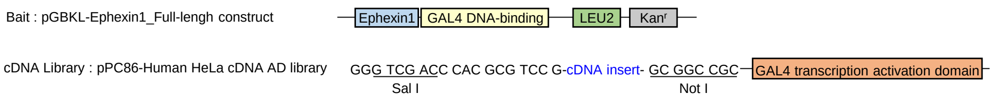

b

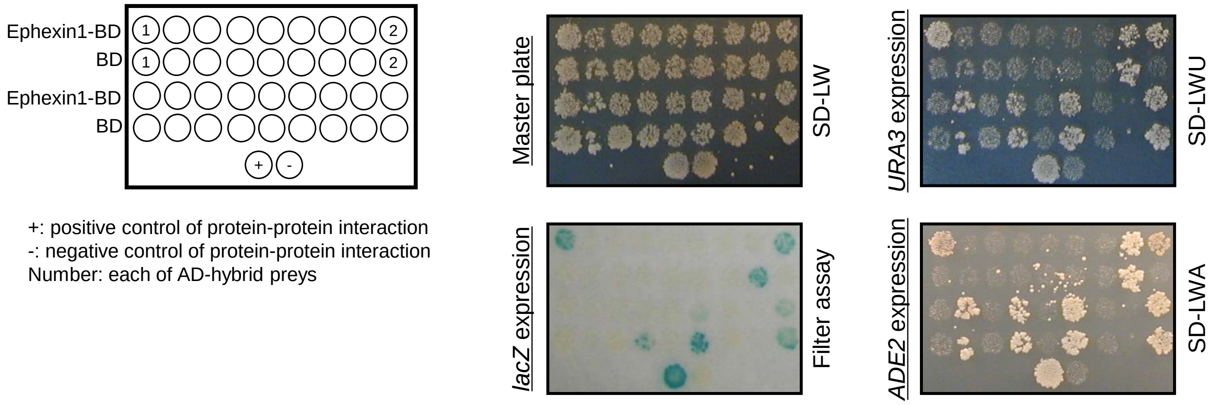

c

Prey ID	Description	Reporter expression		
		lacZ	*HIS3*	*ADE2*
**AD Hybrid – 1, 2**	The activation domain (AD) is fused in frame to the 501st aa of axin 1 (AXIN1), transcript variant 1 (NM_003502).	+	+	+

d

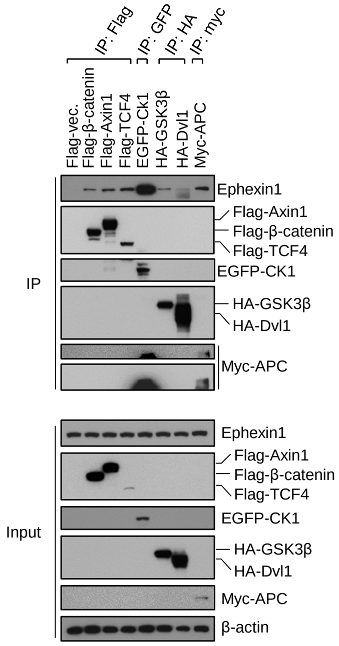

e

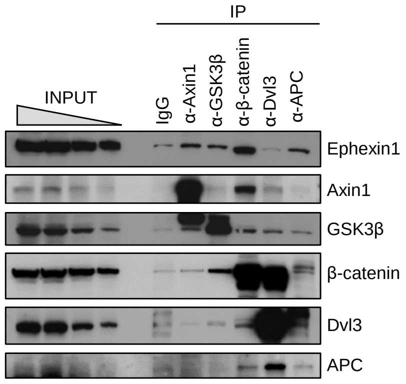

Supplementary Fig. 7 Ephexin1 interacts with the β-catenin destruction complex. a Schematic representation of the bait plasmid (pGBKL-Ephexin1-GAL4-DNA binding domain) and the prey plasmid (GAL4 transcription activation domain-fused HeLa cDNA library). **b** Transformed yeast cells were selected on the minimal media lacking leucine and tryptophan (SD-LW) to select for the bait and prey plasmids, respectively. Specific interactions between the two proteins were monitored by (i) the appearance of a visible blue color in the filter assay; (ii) the growth of colonies on the selective medium lacking leucine, tryptophan, and uracil (SD-LWU); and (iii) the growth of colonies on the selective medium lacking leucine, tryptophan, and adenine (SD-LWA). **c** A list of proteins identified in the screening that interacted with Ephexin1. **d** HEK-293T cells were transfected with Flag-β-catenin, Flag-Axin1, Flag-TCF4, EGFP-CK1, HA-GSK3β, HA-Dvl1, and Myc-APC. Co-immunoprecipitation was performed using the indicated antibodies. The levels of indicated proteins in the extracts and Co-IP products were analyzed by western blot analysis. **e** Extracts from HCT116 cells were subjected to immunoprecipitation using the indicated antibodies. The immunocomplexes and inputs were analyzed by western blot using the indicated antibodies.

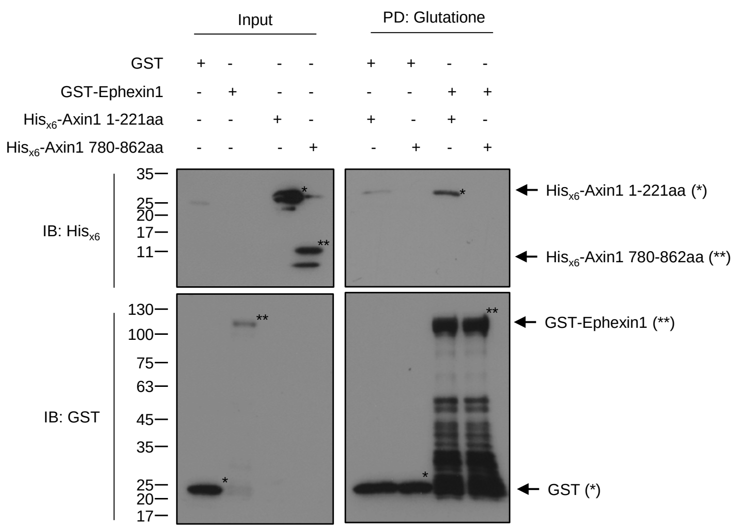

Supplementary Fig. 8 Ephexin1 binds directly to the RGS domain of Axin1. An *in vitro* GST-pulldown assay was conducted to analyze the binding of recombinant Hisx6-tagged Axin1 (1-221aa or 780-862aa) with GST or GST-Ephexin1 full-length. Glutathione beads were used for the GST pulldown assay and were subjected to western blot analysis with His and GST antibodies.

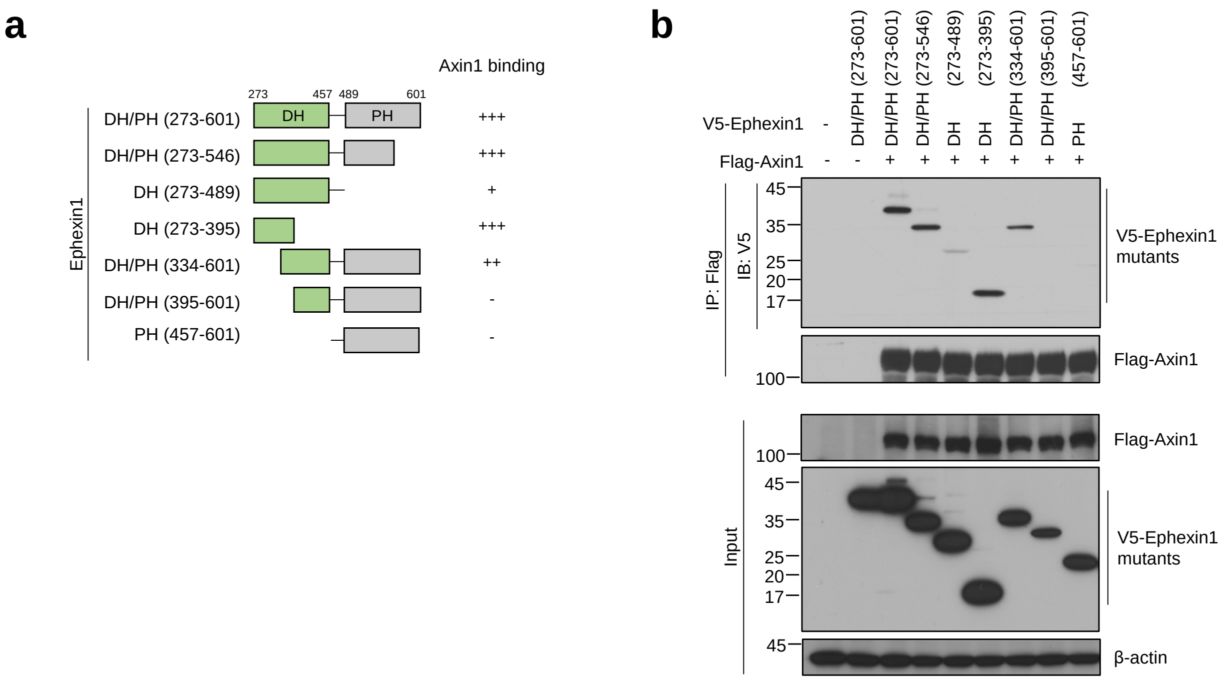

Supplementary Fig. 9 Interaction with the DH domain of Ephexin1 is required for its interaction with Axin1. a Schematic representation of a series of deletion mutants of Ephexin1 is provided. A summary of the binding intensity between each of the Ephexin1 deletion mutants and Axin1 is displayed on the right. **b** Protein extracts from HEK293T cells, co-transfected with V5-tagged Ephexin1 mutants and Flag-tagged Axin1, were immunoprecipitated with an anti-Flag antibody and subjected to western blot analysis using the indicated antibodies.

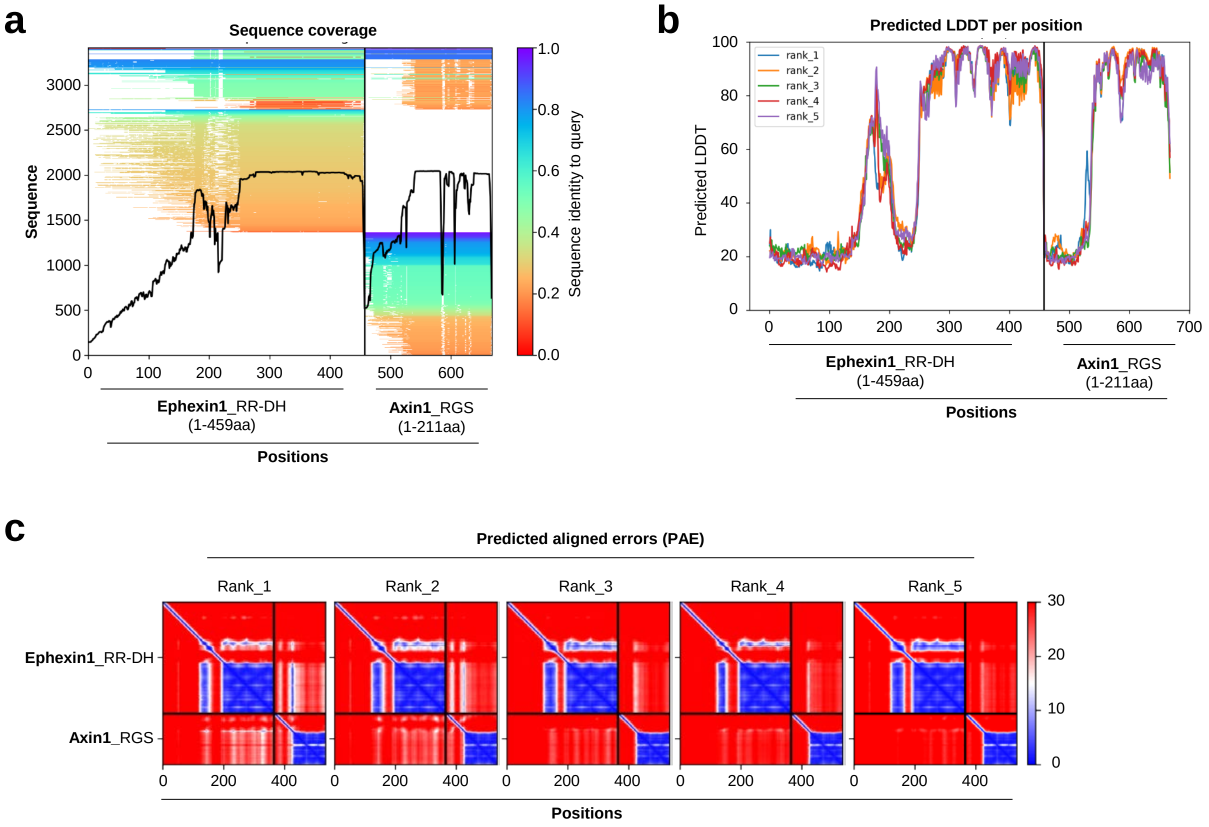

Supplementary Fig. 10 Prediction of the RR-DH domain of Ephexin1 and complex using AlphaFold Multimer. a Sequence coverage values are presented for the RR-DH domain of Ephexin1 and the RGS domain of Axin1 protein predictions. **b** Prediction IDDT (Isolated Distance Difference Test) scores for models are ranked in the Ephexin1 (RR-DH domain) and Axin1 (RGS domain) protein complex prediction. **c** Predictive Alignment Error (PAE) for each ranked model by AlphaFold Multimer. The best model is Ranked 1, and the closer it is to blue, the higher the predicted domain formation and interaction between the two proteins.

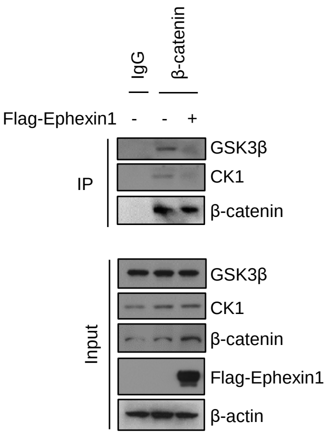

Supplementary Fig. 11 Overexpression of Ephexin1 reduces the interaction between β-catenin and destruction complex proteins. Co-immunoprecipitation analysis was conducted on HEK293T cells transfected with Flag-tagged Ephexin1. Cell lysates were immunoprecipitated with an anti-β-catenin antibody and subjected to western blot analysis with the indicated antibodies.

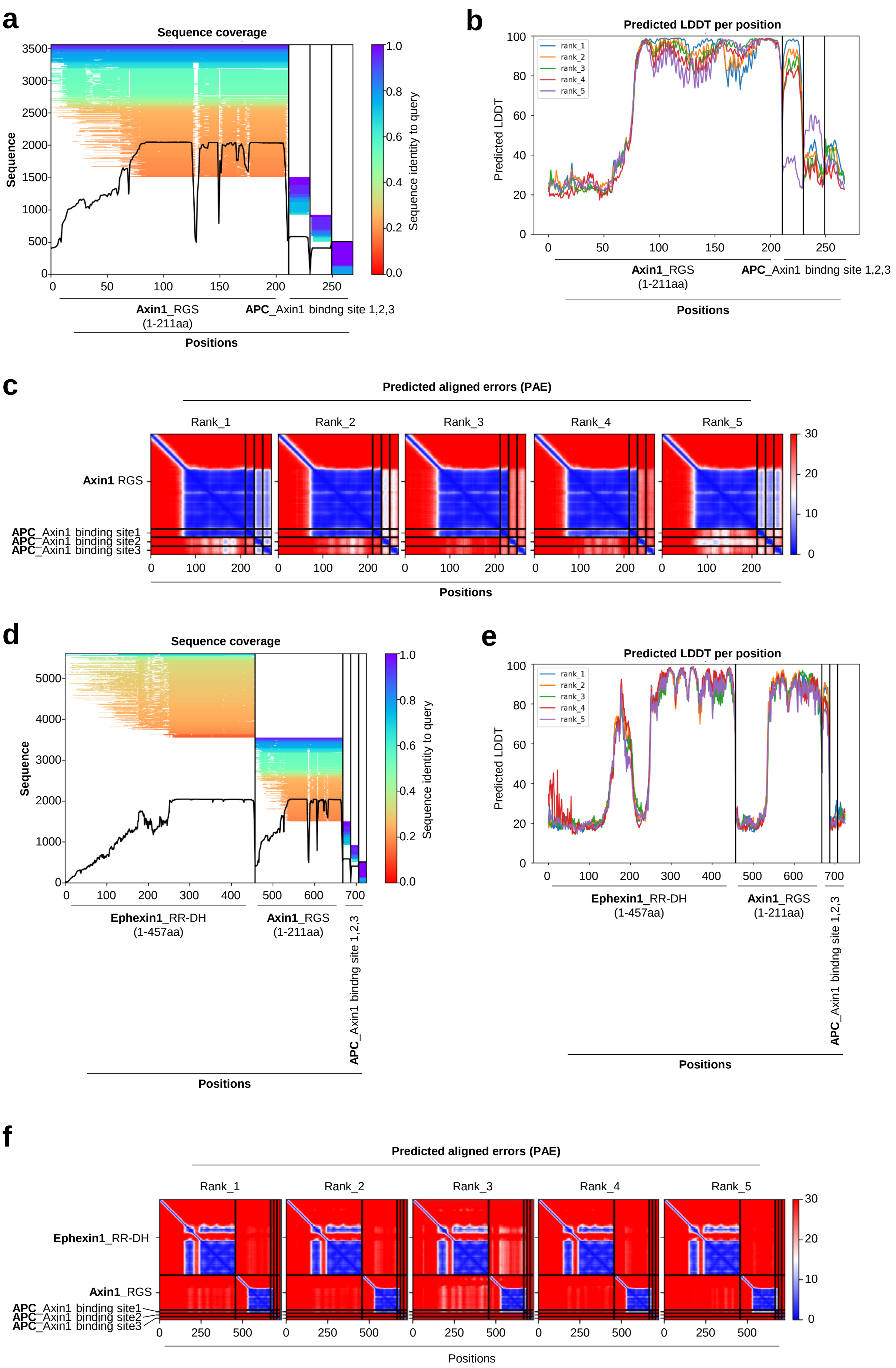

a
Sequence coverage
Sequence
Sequence identity to query
Axin1_RGS (1-211aa)
APC_Axin1 bindng site 1,2,3
Positions
b
Predicted LDDT per position
Predicted LDDT
rank_1
rank_2
rank_3
rank_4
rank_5
Axin1_RGS (1-211aa)
APC_Axin1 bindng site 1,2,3
Positions
c
Predicted aligned errors (PAE)
Rank_1
Rank_2
Rank_3
Rank_4
Rank_5
Axin1 RGS
APC_Axin1 binding site1
APC_Axin1 binding site2
APC_Axin1 binding site3
Positions
d
Sequence coverage
Sequence
Sequence identity to query
Ephexin1_RR-DH (1-457aa)
Axin1_RGS (1-211aa)
APC_Axin1 bindng site 1,2,3
Positions
e
Predicted LDDT per position
Predicted LDDT
rank_1
rank_2
rank_3
rank_4
rank_5
Ephexin1_RR-DH (1-457aa)
Axin1_RGS (1-211aa)
APC_Axin1 bindng site 1,2,3
Positions
f
Predicted aligned errors (PAE)
Rank_1
Rank_2
Rank_3
Rank_4
Rank_5
Ephexin1_RR-DH
Axin1_RGS
APC_Axin1 binding site1
APC_Axin1 binding site2
APC_Axin1 binding site3
Positions

Supplementary Fig. 12 Prediction of Ephexin1 (RR-DH domain), Axin1 (RGS domain), and APC (Axin1 binding site1,2,3) protein complex using AlphaFold-Multimer. a Sequence coverage values for predictions related to RGS domain of Axin1 (1-211aa) and the three Axin1 binding sites on APC: site 1 (1567-1595aa), site 2 (1716-1734aa), and site 3 (2032-2050aa). **b** Prediction IDDT (Isolated Distance Difference Test) scores for models ranked in the Ephexin1 (RR-DH domain) and Axin1 (RGS domain) protein complex prediction. **c** Predicted alignment errors (PAE) for models of Axin1 and APC by AlphaFold Multimer. **d** Sequence coverage values for predictions related to RR-DH domain of Ephexin1, RGS domain of Axin1, and three A xin1 binding sites of APC (sites 1, 2, 3). **e** Prediction IDDT scores for models ranked in the Ephexin1 / Axin1 / APC protein complex prediction. **f** Predicted alignment errors (PAE) for models of Ephexin1 (RR-DH domain), Axin1 (RGS domain), and APC (Axin1 binding site1,2,3) by AlphaFold-Multimer.

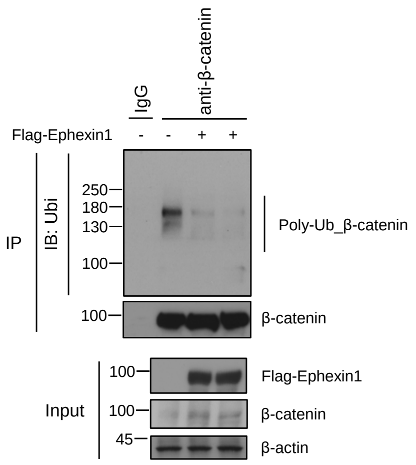

Supplementary Fig. 13. Ephexin1 overexpression inhibits ubiquitination of β-catenin. Lysates from Flag-vector and Flag-Ephexin1 HCT116 cells were immunoprecipitated with an anti-β-catenin antibody and subjected to western blot analysis with the indicated antibodies.

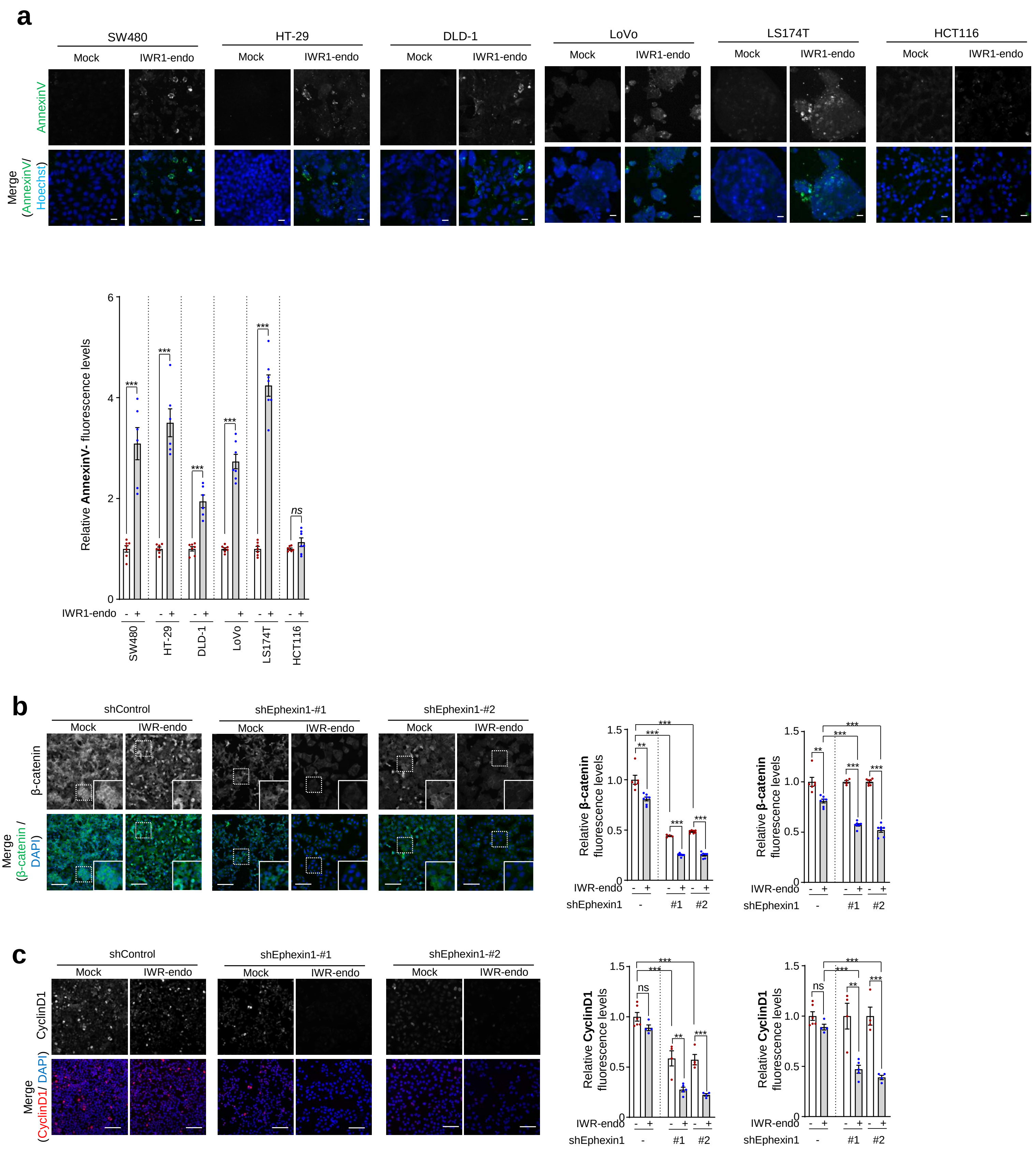

a
SW480
HT-29
DLD-1
LoVo
LS174T
HCT116
Mock
IWR1-endo
AnnexinV
Merge (AnnexinV/ Hoechst)
Relative AnnexinV- fluorescence levels
***
ns
IWR1-endo
b
shControl
shEphexin1-#1
shEphexin1-#2
Mock
IWR-endo
β-catenin
Merge (β-catenin / DAPI)
Relative β-catenin fluorescence levels
**
***
IWR-endo
shEphexin1
#1
#2
c
CyclinD1
Merge (CyclinD1/ DAPI)
Relative CyclinD1 fluorescence levels
ns

d

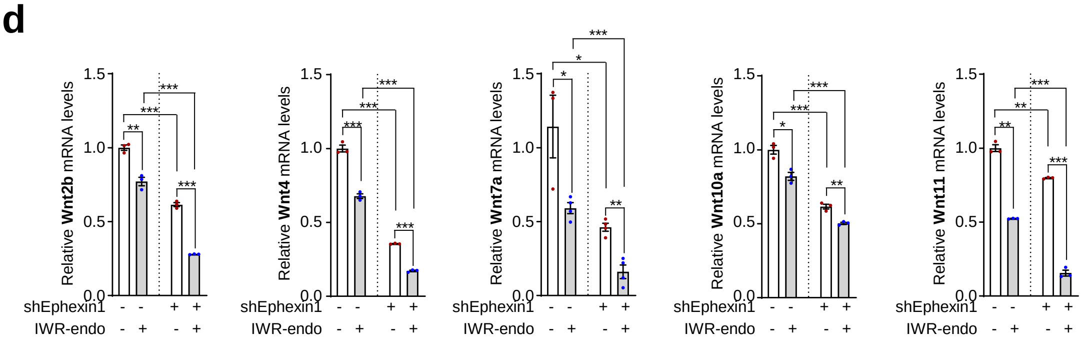

Supplementary Fig. 14. Effects of Wnt/β-catenin signaling inhibition by IWR1-endo treatment in CRC cell lines. a The indicated CRC cell lines (SW480, HT-29, DLD-1, LoVo, LS174T, and HCT116) were either treated with IWR1-endo (80 μM) or left untreated for 12 hours, followed by staining with Annexin V (green) for immunohistochemical analysis. Data are presented as mean ± SEM. ns, not significant; ***p < 0.001, two-tailed Student's t-test. **b, c** Control and Ephexin1-deficient HCT116 cells were either treated with IWR1-endo (80 μM) or left untreated for 12 hours, and then labeled with β-catenin (**b**) and cyclin D1 (**c**) for immunohistochemical analysis. Data are presented as mean ± SEM. ns, not significant; **p < 0.01; ***p < 0.001, two-tailed Student's t-test. **d** The mRNA levels of Wnt ligand in Control and Ephexin1-deficient HCT116 cells were analyzed by qRT-PCR analysis.

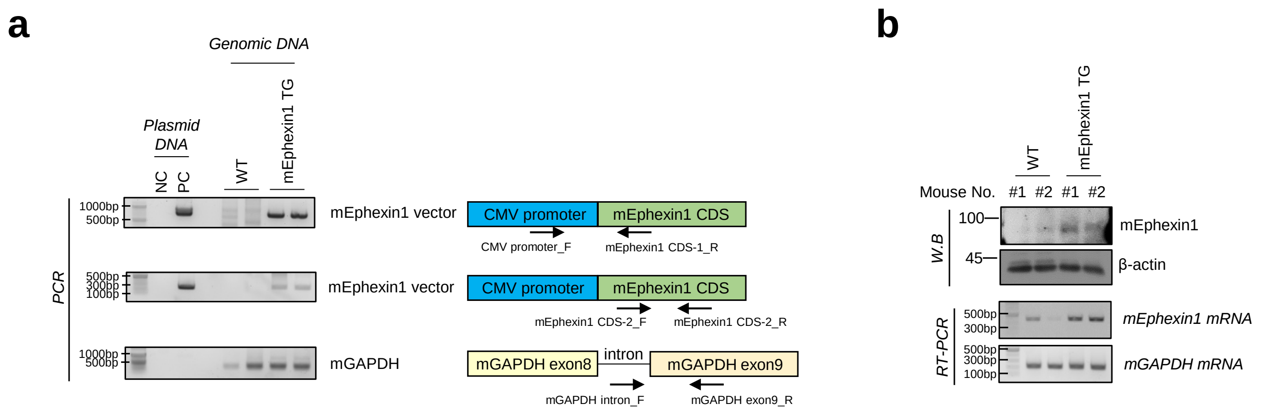

Supplementary Fig. 15 Testing Ephexin1 Transgenic (TG) Mice. **a** PCR analysis was performed on wild-type (WT) and Ephexin1 TG mice using the primer pairs shown in the adjacent panels. Arrows indicate the locations of PCR primers. The sequences of the primer set for detecting the Ephexin1 expression vector (CMV promoter (forward) and mEphexin1 CDS-1 (reverse); mEphexin1 CDS-2 (forward) and mEphexin1 CDS-2 (reverse)) are provided in the ‘Methods’ section. **b** Western blot and RT-PCR analyses of wild-type (WT) and Ephexin1 TG mice were conducted.

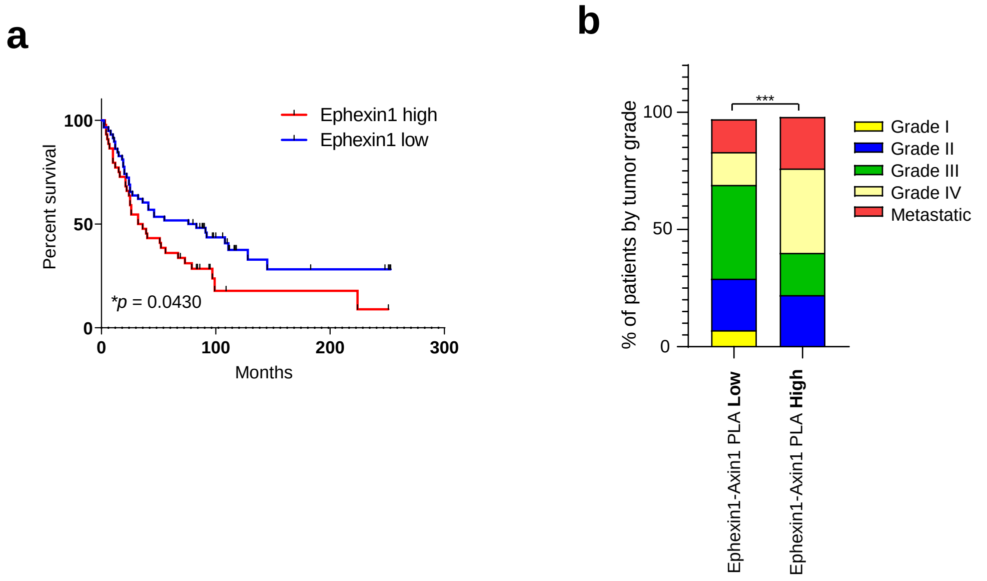

Supplementary Fig. 16. The interaction between Ephexin1 and Axin1 is associated with the prognosis and tumor grade of colorectal cancer patients. a Kaplan-Meier graph showing overall survival of patients according to Ephexin1 expression level in CRC. **b** Stacked bar graph of tumor grade in CRC patients according to Ephexin1-Axin1 PLA score level. ***$p < 0.001$. *P* values are for Chi-square test.

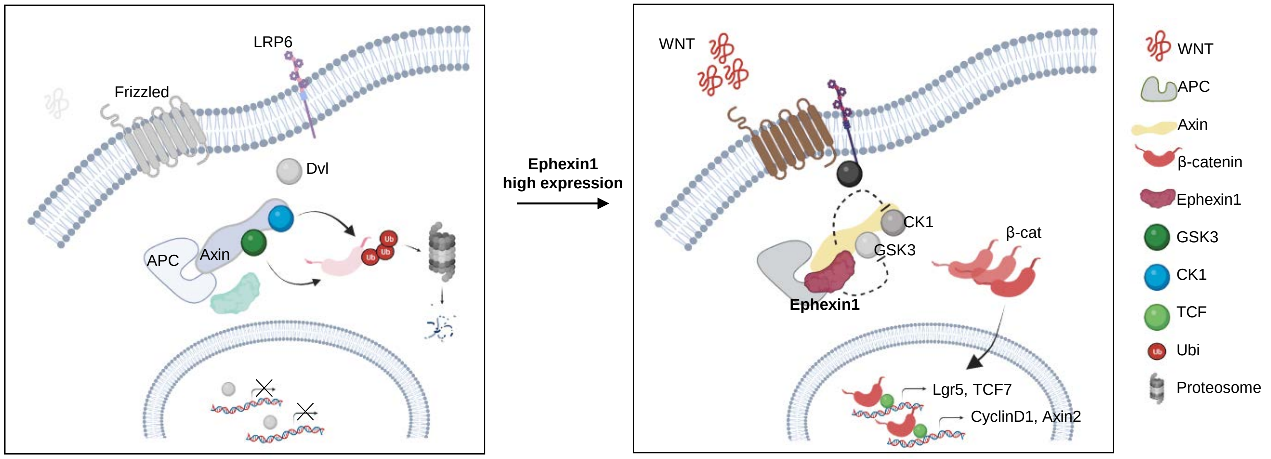

Supplementary Fig. 17 Model of Ephexin1-mediated Wnt/β-catenin signaling regulation. Schematic representation of the model illustrating the primary mechanism of the tumorigenic effect of Ephexin1-mediated Wnt/β-catenin signaling in CRC.

Supplementary Table 1. List of primer sequences for cloning.

Genes		Forward primer sequence	Reverse primer sequence
pET28a-Hisx6-Axin1 (1-211aa)		5'-AAAAGAATTCATGAATATCCAAGAGCAGGGTTTC-3'	5'-AAAAGCGGCCGCTCACGTATATTCCAAATAAATATCAGACTTAAG-3'
pET28a-Hisx6-Axin1 (780-862aa)		5'-AAAAGAATTCGACAGCATCGTTGTGGCGTAC-3'	5'-AAAAGCGGCCGCTCAGTCCACCTTCTCCACTTTG-3'
pCI-Flag-Axin1 Full-length		5'-AAAACTCGAGATGAATATCCAAGAGCAGGGTTC-3'	5'-TTTTGCGGCCGCTCAGTCCACCTTCTCCACTTTG-3
pCI-Flag-Axin1 ΔRGS		5'-AAAACTCGAGATGAGGACAGGCTCGGAGAGCC-3'	5'-TTTTGCGGCCGCTCAGTCCACCTTCTCCACTTTG-3
pCI-Flag-Axin1 ΔRGS/p53		5'-AAAACTCGAGATGAGGATCCGTAAGCAGCACCG-3'	5'-TTTTGCGGCCGCTCAGTCCACCTTCTCCACTTTG-3
pCI-Flag-Axin1 ΔRGS/p53/GSK3β		5'-AAAACTCGAGATGCCGTGTCACAAGCTGCCTC-3'	5'-TTTTGCGGCCGCTCAGTCCACCTTCTCCACTTTG-3
pCI-Flag-Axin1 RNF11/DIX		5'-AAAACTCGAGATGCCAGTGGCACTGGGG-3'	5'-TTTTGCGGCCGCTCAGTCCACCTTCTCCACTTTG-3
pCI-Flag-Axin1 ΔDIX		5'-AAAACTCGAGATGAATATCCAAGAGCAGGGTTC-3'	5'-AAAAGCGGCCGCTCAACACGGCTGGGCACTCC-3'
pCI-Flag-Axin1 RNF11		5'-AAAACTCGAGATGCCAGTGGCACTGGGG-3'	5'-AAAAGCGGCCGCTCAACACGGCTGGGCACTCC-3'
pCI-Flag-Axin1 p53		5'-AAAACTCGAGATGAGGACAGGCTCGGAGAGCC-3'	5'-AAAAGCGGCCGCTCAGTATGGGGGGATCCCATCC-3'
pCI-Flag-Axin1 RGS		5'-AAAACTCGAGATGAATATCCAAGAGCAGGGTTC-3'	5'-AAAAGAATTCTCACGTATATTCCAAATAAATATCAGACTT-3'
pCI-Flag-Axin1 Δp53	Fragment1	5'-AAAACTCGAGATGAATATCCAAGAGCAGGGTTC-3'	5'-AAAAGAATTCCGTATATTCCAAATAAATATCAGACTT-3'
	Fragment2	5'-AAAAGAATTCATGAGGATCCGTAAGCAGCACCG-3'	5'-TTTTGCGGCCGCTCAGTCCACCTTCTCCACTTTG-3
pCI-Flag-Axin1 ΔRNF11/DIX		5'-AAAACTCGAGATGAATATCCAAGAGCAGGGTTC-3'	5'-AAAAGAATTCTCACTTGGCCACGTGCCCACT-3'
pCI-Flag-Axin1 ΔRNF11	Fragment1	5'-AAAACTCGAGATGAATATCCAAGAGCAGGGTTC-3'	5'-AAAAGAATTCCTTGGCCACGTGCCCACT-3'
	Fragment2	5'-AAAAGAATTCGACAGCATCGTTGTGGCGTAC-3'	5'-TTTTGCGGCCGCTCAGTCCACCTTCTCCACTTTG-3
pCI-Flag-Axin1 GSK3β/β-catenin		5'-AAAACTCGAGATGAGGATCCGTAAGCAGCACCG-3'	5'-AAAAGAATTCCTTGGCCACGTGCCCACT-3'
pCI-Flag-Axin1 GSK3β/β-catenin/DIX	Fragment1	5'-AAAACTCGAGATGAGGATCCGTAAGCAGCACCG-3'	5'-AAAAGAATTCCTTGGCCACGTGCCCACT-3'
	Fragment2	5'-AAAAGAATTCGACAGCATCGTTGTGGCGTAC-3'	5'-TTTTGCGGCCGCTCAGTCCACCTTCTCCACTTTG-3
pCI-Flag-Axin1 GSK3β/β-catenin/DIX	Fragment1	5'-AAAACTCGAGATGAATATCCAAGAGCAGGGTTC-3'	5'-AAAACTCGAGCGTATATTCCAAATAAATATCAGACTT-3'
	Fragment2	5'-AAAACTCGAGATGAGGATCCGTAAGCAGCACCG-3'	5'-AAAAGAATTCCTTGGCCACGTGCCCACT-3'
	Fragment3	5'-AAAAGAATTCGACAGCATCGTTGTGGCGTAC-3'	5'-TTTTGCGGCCGCTCAGTCCACCTTCTCCACTTTG-3

Supplementary Table 2. List of primer sequences for RT-qPCR.

Genes	Forward primer sequence	Reverse primer sequence
Ephexin1	5'-GAGATGAAGCGTTGGATGACC-3'	5'-GCCTCTCTCCTGGTCGTGC-3'
Wnt7a	5'-CCCGGACTCTCATGAACTTG-3'	5'-ACGGCCTCGTTGTACTTGTC-3'
Axin2	5'-AGTCAGCAGAGGGACAGGAA-3'	5'-AGCTCTGAGCCTTCAGCATC-3'
CXCL8	5'-CTGCGCCAACACAGAAATTA-3'	5'-GCTTGAAGTTTCACTGGCATC-3'
TERT	5’-CGGTGTGCACCAACATCTACA-3'	5’-GTGTCGAGTCAGCTTGAGCA-3'
YWHAB	5'-ACCCAATTCGTCTTGGTCTG-3'	5'-TCCGATGTCCACAGAGTGAG-3'
APC	5'-CAAAGTCCTAAGCGCCATTC-3'	5'-TGGCTTCCAGAACAAAAACC-3'
DKK1	5'-TCCGAGGAGAAATTGAGGAA-3'	5'-CCTGAGGCACAGTCTGATGA-3'
TCF7	5'-AGAAGAAGAGGCGGTCGAGG-3'	5'-AGCAGATGGTATGAGGGTG-3'
LGR5	5'-AGTGCTGTGCATTTGGAGTG-3'	5'-AGGGCTTTCAGGTCTTCCTC-3'
Wnt9a	5'-GACGGTCAAGCAAGGATCTG-3'	5'-TGCCGTCTCATACTTGTGCT-3'
ID2	5'-CTGGACTCGCATCCCACTAT-3'	5'-CACACAGTGCTTTGCTGTCA-3'
CSNK2B	5'-CCTACTTCGGCACTGGTTTC-3'	5'-GACTGGGCTCTTGAAGTTGC-3'
PPP3CA	5'-TTCCATTTGTTGGGGAAAAA-3'	5'-TTGCCTATTGCTCGGATCTT-3'
CyclinD1	5'-CTGGCCATGAACTACCTGGA-3'	5'-GTCACACTTGATCACTCTGG-3'
CHD1	5'-AAGAAGGAGGCGGAGAAGAG-3'	5'-GCCGCTTTCAGATTTTCATC-3'
ROCK2	5'-GGGTTAGTCGGTTGGTGAAA-3'	5'-TTGGGCCATCATATTTCAGTC-3'
XPO1	5'-GTGACAGGGCTTTTCAGCTT-3'	5'-GATGCCAGGGACAGACATTT-3'
YWHAZ	5'-ACTGGGTCTGGCCCTTAACT-3'	5'-CCGATGTCCACAATGTCAAG-3'
FRAT2	5'-CTGGTGAAGGTGGGAGATGT-3'	5'-CAACAGGGCTCTTCTTGGAG-3'
TBL1XR1	5'-TTTCAGCATTCGTTGCTTTG-3'	5'-GCACAAAACCATCGTGAATG-3'
PRKACB	5'-CACGGTTCTATGCAGCTCAG-3'	5'-CTAATGCCCACCAATCCACT-3'
HDAC1	5'-ACGAATTGCCTGTGAGGAAG-3'	5'-GCTTCTGGCTTCTCCTCCTT-3'
β-actin	5’-CATCCGCAAAGACCTGTA-3’	5’-AGGGTGTAACGCAACTAAG-3’

Supplementary Table 3. List of antibodies

Antigen	Host	Source	Dilution	Cat. No	Applications
Ephexin1	Rabbit	Abcam	1:1000	ab157593	Western blot
Ephexin1	Rabbit	Thermo Scientific	1:100	PA5-52521	IHC & PLA
β-catenin	Mouse	BD	1:2000, 2ug, 1:200, 1:200	#610154	WB & IP & IHC & ICC
Lgr5	Mouse	Thermo Scientific	1:1000	MA5-25644	WB & IHC
β-actin	Mouse	Abcam	1:3000	ab6276	Western blot
Ki67	Mouse	Cell signaling	1:100	#9449	IHC
Phospho-β-catenin Thr41, Ser45	Rabbit	Cell signaling	1:500	#9565	Western blot
Active-β-catenin	Mouse	Millipore	1:500	#05-665	Western blot
C-myc	Mouse	Santa Cruz	1:250, 2ug	SC-40	WB & IP
CyclinD1	Rabbit	Cell signaling	1:1000, 1:100	#2978	WB & ICC
Flag	Mouse	Sigma-Aldrich	1:3000	F1804	WB & IP
Axin1	Rabbit	Cell signaling	1:1000, 2ul	#3323	WB & IP
Axin1	Mouse	LSBio	1:50	LS-C682465	PLA
APC	Mouse	Santa Cruz	1:250, 2ug	SC-9998	WB & IP
GSK3β	Rabbit	Cell signaling	1:1000, 2ul	#9315	WB & IP
V5	Mouse	Invitrogen	1:3000, 2ug	R960-25	WB & IP
Phopho-LRP6 Ser1490	Rabbit	Cell signaling	1:1000	#2568	Western blot
His	Mouse	Abcam	1:500	ab18184	Western blot
GST	Mouse	Santa Cruz	1:500	SC-138	Western blot
GFP	Mouse	Santa Cruz	1:500, 2ug	SC-9996	WB & IP
HA	Rabbit	Santa Cruz	1:500, 2ug	SC-805	WB & IP
Dvl3	Mouse	Santa Cruz	1:250, 2ug	SC-8027	WB & IP
CK1	Mouse	Abcam	1:2000, 2ug	ab82426	WB & IP
Ubi	Mouse	Santa Cruz	1:500	SC-8017	Western blot
